# Pediatric hearing loss in the universal newborn hearing screening era: etiologies, risk indicators, and clinical phenotypes

**DOI:** 10.3389/fpubh.2026.1836340

**Published:** 2026-09-24

**Authors:** Ziyuan Zhao, Yaxuan Wang, Hua Qiu, Xiaofang Sui, Yiying Zhang

**Affiliations:** 1 School of Public Health, Jiamusi University, Jiamusi, China; 2 School of Clinical Medicine, Jiamusi University, Jiamusi, China; 3 Department of Geriatric, The First Affiliated Hospital of School, Jiamusi University, Jiamusi, China; 4 Department of Epidemiology and Biostatistics, School of Public Health, Jiamusi University, Jiamusi, China

**Keywords:** congenital cytomegalovirus infection, genetic etiology, pediatric hearing loss, risk indicators, universal newborn hearing screening

## Abstract

Childhood hearing loss poses significant developmental and public health challenges, impacting language acquisition, cognitive development, social participation, and educational outcomes. Universal newborn hearing screening (UNHS) has notably enhanced the early identification of congenital hearing loss, yet limitations persist. Mild, unilateral, delayed-onset, progressive, and certain neural forms of hearing loss may remain undetected during a single neonatal screening session. This review consolidates current evidence regarding the etiologies, risk indicators, and clinical features of pediatric hearing loss in the context of UNHS. Essential areas of focus encompass genetic causes and pharmacogenetic susceptibility, congenital cytomegalovirus infection, intrauterine nutritional and metabolic conditions, environmental and lifestyle exposures, alongside perinatal or neonatal intensive care unit-related risks such as prematurity, hyperbilirubinemia, hypoxia, infection, and exposure to ototoxic medications. These factors exhibit varied clinical implications: some serve as direct causes of hearing loss, while others signify heightened vulnerability to delayed-onset or progressive auditory dysfunction. The evidence highlights a developmental perspective on pediatric hearing loss, indicating that auditory risks may emerge postnatally and cannot be ruled out based solely on normal newborn screening results. Distinguishing between etiology, risk indicators, and clinical phenotype may enhance the identification of children requiring intensified post-screening monitoring. Future research should aim to refine causal evidence, genotype–phenotype correlations, and viable strategies for risk-based follow-up.

## 1 Introduction

Childhood hearing loss represents a major developmental and public health concern. Its consequences extend beyond reduced auditory sensitivity and include delayed language acquisition, impaired communication, altered cognitive development, reduced social participation, emotional difficulties, and educational disadvantage (1–3). Infancy and early childhood are critical periods for auditory pathway maturation and language-related neural development. Therefore, prolonged or unrecognized auditory deprivation during these stages may exert lasting effects on speech, learning, and psychosocial functioning. The burden is particularly substantial in low- and middle-income settings, where limited access to screening, diagnostic confirmation, amplification, cochlear implantation, and rehabilitation may further exacerbate developmental inequalities (4, 5).

The implementation of universal newborn hearing screening (UNHS) has altered early detection of congenital hearing loss. Based on objective physiological measures such as otoacoustic emissions (OAE) and automated auditory brainstem response (AABR), UNHS has lowered the age of diagnosis and enhanced access to early intervention in numerous health systems (6, 7). Nevertheless, the limitations of a single neonatal screening episode have become increasingly apparent. Current screening protocols are effective for detecting many congenital moderate-to-profound hearing loss cases present at birth. However, they are less sensitive to mild, unilateral, delayed-onset, progressive, and some neural forms of hearing impairment, including auditory neuropathy spectrum disorder (ANSD) (6, 8–10). Therefore, a normal newborn screening result cannot always be considered an absence of long-term hearing risk (Figure 1). At the same time, the causes of hearing loss in children under the UNHS are becoming more complicated. Historically, most childhood hearing loss was caused by obvious congenital defects, serious infections or perinatal injuries. More recent studies have shown that the causes of pediatric hearing loss are more diverse and heterogeneous, including genetic variations with variable penetrance, congenital cytomegalovirus (cCMV) infection, intrauterine nutritional and metabolic disorders, environmental and lifestyle factors, pharmacogenetic susceptibility, and clusters of risk in neonatal intensive care units (NICU) (11–13). They have different uses. Some are direct causes of the disease; others are risk factors or markers of vulnerability that affect the development of the disease. Therefore, the above categories need to be distinguished for both clinical application and research integration.

**Figure 1:**
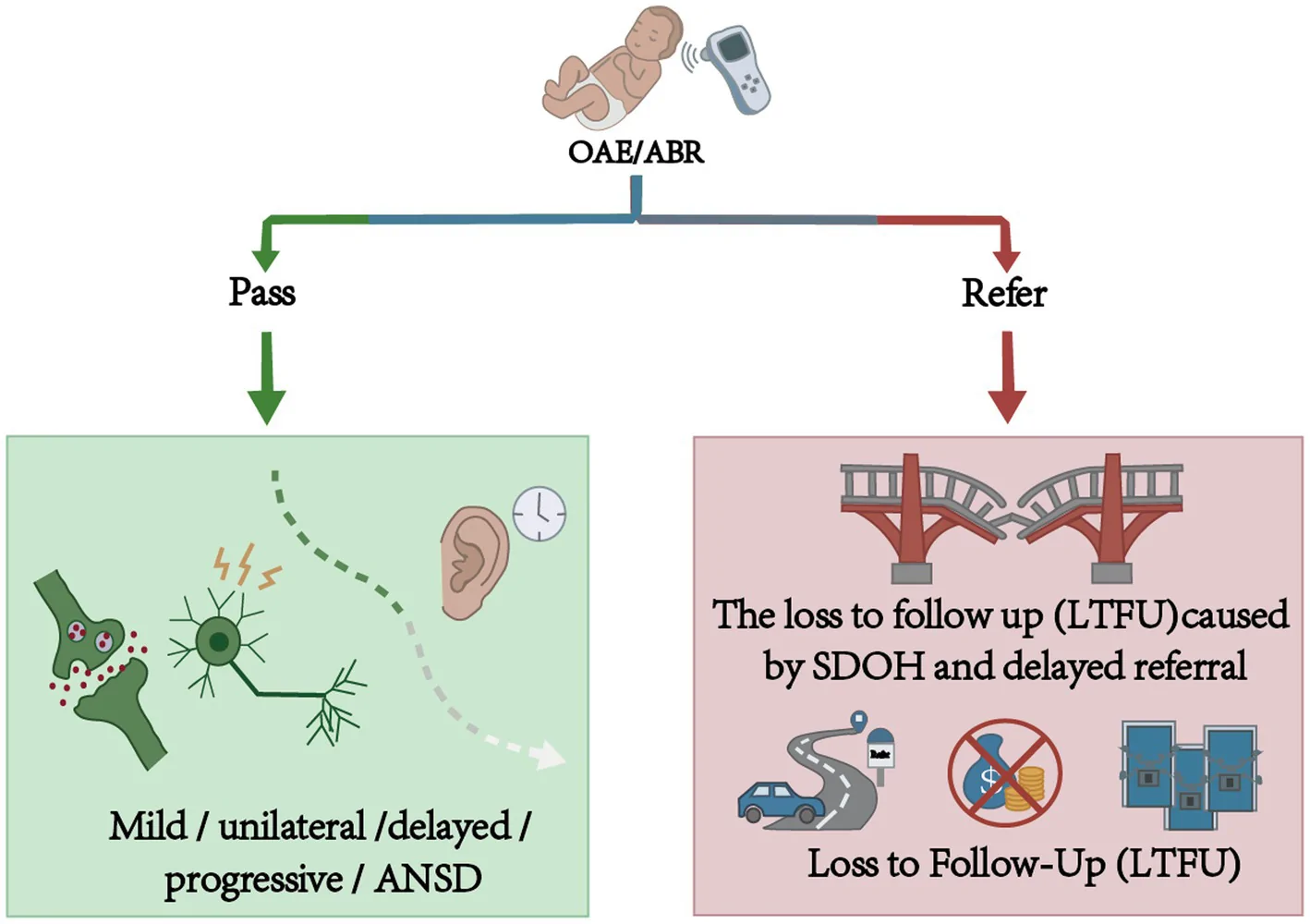
Limitations of universal newborn hearing screening in detecting heterogeneous pediatric hearing loss.

Childhood hearing loss also has many different forms in the clinic. Hearing loss can be congenital, delayed-onset, or progressive; it can be conductive, cochlear, synaptic, neural, or involve the central auditory pathway; and it may present as an isolated condition or as part of a syndrome. All of the above differences will affect how audiological indicators are recognized and interpreted, as well as which children need to be selected for follow-up tests after new-born screening. Specifically, circumstances such as cCMV-related hearing loss, enlarged vestibular aqueduct (EVA), OTOF-related ANSD, mt-RNR1-associated aminoglycoside susceptibility, and NICU-related neural injury indicate that the deficiencies of threshold-based neonatal screening in diagnosing pediatric hearing loss need to be addressed.

Amidst this context, the purpose of this review is to synthesize evidence concerning the major etiologies, risk indicators, and clinical features of pediatric hearing loss in the UNHS era. Focus areas include genetic causes, infectious etiologies, intrauterine and maternal–infant exposures, and perinatal or NICU-related risks, with emphasis on their implications for delayed-onset and progressive hearing loss. Additionally, how temporal trajectory, lesion level, and audiological phenotype influence detection and assessment is summarized. By elucidating the distinctions between etiology, risk indicators, and clinical phenotype, this review aims to offer a structured foundation for risk-informed surveillance without advocating for a comprehensive clinical management guideline.

## 2 Concepts and classification

### 2.1 Temporal trajectories of childhood hearing loss: congenital, delayed-onset, and progressive forms

Classifying childhood hearing loss by its temporal trajectory is critical for effective clinical surveillance and public health management. Congenital hearing loss, characterized by auditory impairment present at birth, is typically identified through objective audiological screening during the neonatal period, thereby representing the primary focus of UNHS. In contrast, delayed-onset and progressive hearing loss exhibit greater temporal heterogeneity: affected children may initially exhibit normal or near-normal hearing but subsequently experience deterioration over months or years, potentially extending into the preschool years, or may present with mild initial abnormalities that worsen over time (14). These forms constitute a significant source of missed diagnoses in current childhood hearing loss prevention programs, as they often remain undetectable during a single neonatal screening encounter.

This temporal heterogeneity carries direct implications for screening strategies and follow-up designs. For congenital hearing loss, the emphasis lies on early diagnosis and timely intervention within the critical period for language development. Conversely, delayed-onset and progressive forms differ from congenital hearing loss due to their auditory phenotypes not being apparent during the neonatal period. This variability is clinically relevant, elucidating why some children who pass UNHS may later exhibit hearing deterioration. Previous studies have identified that certain genetic forms of hearing loss follow characteristic age-dependent trajectories. For instance, STRC-associated autosomal recessive hearing loss may progress slowly yet persistently over time (15). Similarly, pediatric unilateral sensorineural hearing loss (USNHL) and idiopathic sudden sensorineural hearing loss (ISSNHL) demonstrate that hearing loss may manifest at varying stages of childhood and may progress further or impact the contralateral ear (10, 16, 17). These patterns highlight the notion that childhood hearing loss should be regarded as a dynamic developmental process rather than a static diagnosis.

The deficiencies in UNHS are thus more evident. Newborn hearing screening is inherently a cross-sectional assessment conducted at a single time point; it can identify hearing loss present at birth above a certain threshold but lacks the ability to predict the later development of delayed-onset or progressive hearing impairment. Therefore, a normal result of the newborn screen does not guarantee a low long-term risk. Temporal classification refers to the requirement for follow-up care after the neonatal period for infants with genetic susceptibilities, intrauterine infections, NICU exposure, or a family history of positive cases. Thus, the strategy of prevention and management should extend beyond the time of birth to include continuous risk evaluation during early life (14). In practice, time-based division of the population is used to allocate more resources to high-risk groups, such as newborns with suspected congenital hearing loss and older children or adults with delayed onset hearing loss and progressive hearing loss, requiring repeated monitoring and intervention (Table 1).

**Table 1:** Temporal trajectories of childhood hearing loss and corresponding clinical management strategies.

Trajectory type	Definition and time of occurrence	Screening features and challenges	Key prevention and management strategies
Congenital hearing loss	Hearing impairment is present at birth.	This is the primary target of UNHS. Objective single-visit neonatal screening can usually identify affected infants during the newborn period.	Emphasize early confirmation, timely referral, rehabilitation, and hearing-aid fitting or other interventions within the critical window for language development.
Delayed-onset hearing loss	Hearing thresholds are normal or near-normal at birth, but hearing loss develops months or years later, often before school age.	A single normal UNHS result may produce false reassurance because the disorder has marked temporal variability. Therefore, neonatal screening alone cannot exclude later hearing loss.	Establish continuous surveillance based on risk indicators, repeated hearing assessments, parental education, and prompt evaluation when speech, language, or auditory concerns emerge.
Progressive hearing loss	Mild or subclinical hearing impairment is present initially and gradually worsens over time.	Early abnormalities may be subtle or below the screening threshold at birth, but hearing thresholds deteriorate with age. This may limit the sensitivity of one-time neonatal screening.	Develop long-term risk stratification beyond the neonatal period and dynamically adjust follow-up intervals according to the rate and pattern of hearing deterioration.

### 2.2 Peripheral, synaptic, and central auditory levels: lesion hierarchy and phenotypic diversity

The heterogeneity of childhood hearing loss is characterized not only by timing of onset, but also by lesion level and functional phenotype. Broadly, pediatric hearing loss can be classified according to the site of dysfunction within the auditory pathway: peripheral conductive or cochlear lesions, synaptic and auditory nerve disorders, and abnormalities involving central auditory networks. Each level is associated with distinct audiological features and varying limitations in screening detectability.

Peripheral conductive and cochlear lesions are the most prevalent pathological substrates in pediatric hearing loss. These include conductive hearing loss related to outer or middle ear disorders and sensorineural hearing loss (SNHL) resulting from cochlear hair cell dysfunction. Such impairments typically manifest as relatively stable threshold elevations, making them more readily detected using conventional tools such as OAE, acoustic immittance testing, and behavioral audiometry (18). As these lesion types are generally well captured by standard newborn screening and routine audiological follow-up, they represent the phenotypes most easily identified within current prevention and management systems.

However, not all childhood hearing loss originates from outer hair cell or cochlear dysfunction. In certain children, the primary abnormality may occur at the level of inner hair cells, the synapse, or auditory nerve synchrony, with ANSD being the most recognized example. ANSD is characterized by absent or abnormal auditory brainstem responses (ABRs), preserved or partially preserved OAEs, and disproportionate impairment in speech perception relative to pure-tone thresholds (19, 20). Infants screened with OAE alone may be misclassified as low risk during the neonatal period, potentially delaying a definitive diagnosis. The presence of ANSD illustrates that childhood hearing loss cannot be exclusively defined by hearing thresholds; factors such as neural synchrony, temporal processing, and speech recognition must also be considered. Screening and follow-up for these phenotypes should therefore emphasize the combined use of OAE and ABR, along with age-appropriate functional auditory and speech assessments.

In childhood hearing loss, damage can occur in the central auditory pathway and other areas of the brain outside of the cochlea and auditory nerve. Neuroimaging studies have shown that children with congenital severe-to-profound SNHL have abnormal white matter organisation and large-scale connectivity patterns (21). Prolonged hearing loss may be associated with structural changes in the hippocampus, amygdala, and their interconnected covariance networks; thus, in addition to auditory dysfunction, cognition and emotion regulation can be impaired (22). Specific brain areas have also been linked to hearing in children with ANSD (23). Therefore, the above results suggest that childhood hearing impairment should be regarded as a neurodevelopmental disorder with risks of affecting language, working memory, executive functions, and social–emotional development, not simply as an ear problem.

Thus, lesion-based classification can help us learn about the cause and extent of the disease in diagnosis. Peripheral cochlear disorders can be diagnosed relatively well using OAEs, immittance measurements, and pure-tone audiometry; synaptic and neural disorders require electrophysiological methods, such as ABRs, and central involvement must be ruled out by speech perception tests, neurodevelopmental evaluations, and other means such as neuroimaging. In short, childhood hearing loss should be viewed as a spectrum of disorders at all levels of hearing, and corresponding diagnostic plans and follow-up goals are needed for each (Table 2).

**Table 2:** Auditory lesion level, representative pathology, clinical phenotype, and relevant assessment approaches.

Auditory lesion level	Representative pathology or condition	Clinical and audiological features	Relevant assessment approaches
Peripheral conductive or cochlear disorders	Outer or middle ear conductive pathology; cochlear hair-cell dysfunction.	These are common causes of pediatric hearing loss. They usually present with measurable elevation of hearing thresholds and are often detected by universal newborn hearing screening or routine audiological evaluation.	Otoacoustic emissions, acoustic immittance testing, behavioral audiometry, and pure-tone audiometry, according to age and developmental stage.
Synaptic or auditory nerve conduction disorders	Inner hair-cell, synaptic, or auditory nerve dysfunction; auditory neuropathy spectrum disorder.	Otoacoustic emissions may be preserved or partially preserved, whereas auditory brainstem responses are absent or abnormal. Speech perception may be poorer than expected from pure-tone thresholds.	Combined interpretation of otoacoustic emissions and auditory brainstem responses. Speech perception testing and functional auditory assessment may further characterize real-world listening difficulty.
Central auditory pathway or network involvement	Brainstem or central auditory network abnormalities; altered white-matter connectivity; broader neurodevelopmental involvement.	Auditory dysfunction may coexist with difficulties in language, working memory, executive function, emotional regulation, or broader neurodevelopment. Pure-tone thresholds alone may not fully reflect functional impact.	Speech perception testing, age-appropriate neurodevelopmental assessment, central auditory processing evaluation when appropriate, and neuroimaging in selected cases.

### 2.3 Definition of high-risk populations and follow-up windows

Identifying high-risk indicators is important to interpret auditory risks in newborns with normal hearing screening results, as some infants who pass the UNHS may later develop delayed-onset or progressive hearing loss. Therefore, risk stratification should consider all-around data beyond the results of newborn screening and include factors such as genetic predisposition, prenatal exposure, congenital infections, NICU history, craniofacial abnormalities, syndromic features, and family history. According to the current guidelines and long-term cohort studies, the above factors are associated with a higher risk of developing sensorineural hearing loss (6, 14).

Defining a high-risk group to determine how often to follow up and monitor is essential. Delayed-onset hearing loss in infants and young children is often caused by difficulties with language development and other cognitive problems. Without an organized follow-up, the children may not be identified in time and will only present with delayed language acquisition, poor speech recognition or abnormal learning, and communication behavior later on (24). Therefore, follow-up for high-risk children should extend to cover the critical developmental stage of infancy and preschool age, and the frequency of testing should be adjusted based on the severity and number of risk factors. For high-risk infants who are more likely to be severely ill or who have a known pathogenic mutation in cCMV, extended observation should be conducted to assess other aspects of their development.

From a methodological standpoint, follow-up programs for high-risk children should employ age-appropriate tools in a stepwise manner. In early infancy, objective audiological measures such as OAE, ABR, immittance testing, and, when indicated, auditory steady-state response (ASSR) are foundational. As children mature, visual reinforcement audiometry (VRA), conditioned play audiometry (CPA), and speech recognition testing can be progressively integrated. For children with suspected neural conduction abnormalities or discrepancies between hearing thresholds and language performance, further evaluations of central auditory processing and neurodevelopment should also be considered. The ultimate aim of high-risk follow-up is not solely to detect threshold decline, but to identify functional impairments as early as possible during critical stages of neurodevelopment. Such an approach can reduce diagnostic delays, optimize intervention timing, and mitigate the long-term effects of hearing loss on language, cognition, and social functioning (21, 22) (Table 3).

**Table 3:** Definition of high-risk populations and age-progressive follow-up framework.

Evaluation dimension	Core content	Clinical significance and management objective
Definition of high-risk factors	High-risk factors include genetic background, congenital infections such as cytomegalovirus infection, history of neonatal intensive care unit admission, family history of childhood hearing loss, craniofacial anomalies, and syndromic presentations.	Risk-factor identification guides follow-up intervals and monitoring intensity. Importantly, high-risk infants who pass newborn hearing screening may still develop delayed-onset or progressive hearing loss.
Follow-up window	Follow-up should cover the period from infancy to preschool age, which overlaps with rapid development of language, communication, and social cognition.	Follow-up frequency should be dynamically adjusted according to risk severity, with the aim of reducing diagnostic delay and improving the timing of intervention.
Age-appropriate detection tools	In early infancy, objective tests such as otoacoustic emissions, auditory brainstem response, acoustic immittance testing, and auditory steady-state response are recommended. With increasing age, assessment may gradually shift to visual reinforcement audiometry, conditioned play audiometry, and speech recognition testing.	Assessment should move beyond single-threshold detection toward multidimensional evaluation of auditory function and real-world communication ability.
Ultimate management objective	Management should not focus only on detecting deterioration in hearing thresholds, but should also include evaluation of speech development, communication ability, and neurodevelopmental status.	The ultimate goal is to reduce the adverse effects of hearing loss on language, cognitive, social, and emotional development in children.

## 3 Genetic etiology and population diversity

### 3.1 Common non-syndromic deafness genes: GJB2, SLC26A4, OTOF, and mt-RNR1

Genetic factors account for a substantial proportion of permanent childhood hearing loss. Since the widespread implementation of UNHS, their contribution to the overall etiological spectrum has become increasingly apparent (25, 26). Recent studies indicate that non-syndromic hearing loss constitutes the majority of hereditary cases, with a limited number of recurrent genes emerging as key targets for clinical diagnosis and public health screening (25–27). The growing use of multigene panels, whole-exome sequencing (WES), and molecular diagnostic workflows has improved etiological diagnosis and shifted genetic testing from a largely explanatory tool toward an paradigm that can also provide information regarding prognosis, phenotype variability, and medication susceptibility (26, 27).

Among them, GJB2 is one of the most frequent causes of non-syndromic deafness worldwide. The gene codes for connexin 26; this protein regulates potassium recycling and maintains inner-ear homeostasis. GJB2-related hearing loss has a wide range of phenotypes, including congenital severe-to-profound impairment and mild, delayed-onset or progressive forms (28–31). Its public health importance stems from the fact that it is widespread, and some strains have a high carrier rate in the general population and may be associated with children who have passed newborn hearing tests but develop hearing loss later (30–33). To understand the reason for normal or near-normal findings at birth but hearing loss later in childhood, GJB2 should be examined.

SLC26A4 is another high-frequency deafness gene of considerable clinical relevance. This gene encodes pendrin and is closely associated with EVA and Pendred syndrome. Hearing loss linked to SLC26A4 often follows a fluctuating or progressive course and may deteriorate following head trauma, barometric pressure variations, or other physiological stressors (27, 28, 34–36). Its clinical value extends beyond mere etiological confirmation; identifying SLC26A4-related conditions helps elucidate fluctuations in hearing loss and is often interpreted alongside imaging findings indicative of EVA.

OTOF, encoding otoferlin, serves as a representative gene for autosomal recessive ANSD. OTOF-related disease is characterized by relatively preserved outer hair cell function coupled with impaired synaptic transmission, leading to disrupted auditory nerve synchrony. Consequently, affected children may demonstrate preserved OAEs while displaying abnormal ABRs (27). This genotype is clinically significant as it elucidates discrepancies between screening outcomes and auditory performance, while offering useful context for interpreting variations among OAE, ABR, and functional auditory assessments.

The mitochondrial locus mt-RNR1 is a typical case of genetic susceptibility to ototoxicity. It is not a congenital hearing loss but rather a severe sensitivity to aminoglycoside antibiotics; therefore, standard exposure may lead to permanent hearing impairment (27). Most deafness genes are in a recessive mode; mt-RNR1, however, shows incomplete penetrance, and identifying carriers can help avoid lifelong medication use to prevent genetically caused hearing loss. Collectively, the above examples show that the genetic framework for pediatric hearing screening should include both common deafness-causing genes and pharmacogenetic susceptibility loci (Table 4).

**Table 4:** Common non-syndromic hearing-loss genes and their clinical relevance.

Gene	Encoded protein or functional relevance	Common audiological features	Clinical relevance
GJB2	Encodes connexin 26, which is involved in potassium recycling and cochlear homeostasis.	Phenotypes are heterogeneous, ranging from congenital severe-to-profound hearing loss to mild, delayed-onset, or progressive forms.	Genetic findings may help explain variable age of onset and severity. Some affected children may pass newborn hearing screening but develop hearing loss later.
SLC26A4	Encodes pendrin and is commonly associated with enlarged vestibular aqueduct and Pendred syndrome.	Hearing loss is often fluctuating or progressive. Deterioration may occur after minor head trauma, barometric pressure changes, or infection.	Molecular results are clinically useful for interpreting enlarged vestibular aqueduct-associated hearing loss and assessing the risk of progression, particularly when combined with imaging findings.
OTOF	Encodes otoferlin, which is required for inner hair-cell synaptic transmission.	Patients may show an auditory neuropathy spectrum disorder-like phenotype, with preserved or partially preserved otoacoustic emissions and absent or abnormal auditory brainstem responses.	Genetic diagnosis may help explain discrepancies among otoacoustic emissions, auditory brainstem responses, and functional auditory performance.
mt-RNR1	Encodes mitochondrial 12S rRNA and is associated with susceptibility to aminoglycoside ototoxicity.	Hearing may be normal at birth, but aminoglycoside exposure can trigger irreversible sensorineural hearing loss.	Identification of pathogenic variants has pharmacogenetic relevance and may guide avoidance of aminoglycosides when clinically feasible.

### 3.2 Adaptation of screening panels for regional and ethnic heterogeneity

Hereditary hearing loss exhibits significant variation across populations and regions, characterized by marked differences in pathogenic variant spectra, carrier frequencies, and phenotype distribution among countries, ethnic groups, and local populations (26, 34). Fixed screening panels designed for one population may therefore perform suboptimally in another, leading to diagnostic gaps and inefficient resource utilization. The diagnostic yield of genetic screening panels is strongly influenced by their alignment with local molecular epidemiology.

GJB2, SLC26A4, and mt-RNR1 have been frequently included in the screening lists for newborn and childhood hearing loss in the Chinese population; however, the frequency of particular variants and their related symptoms differ across different regions (34, 35, 37, 38). Large-scale studies in eastern and southern China have shown that SLC26A4-related variants and corresponding imaging abnormalities are more frequent in certain areas, and thus the design of regional panels may affect not only the detection rate but also the subsequent priorities for counseling and follow-up, such as EVA surveillance and head injury prevention (34, 35, 37). On the other hand, in areas with high genetic diversity and a lack of historical molecular data, commercial panels developed for European, North American or East Asian populations often show reduced efficacy (39). Many existing hotspot variants are not suitable for explanation in light of these circumstances; therefore, locally derived sequencing data need to inform the panel design.

The same applies to groups of people with a greater risk of inbreeding. Autosomal recessive hearing loss in Palestinians and some other Middle Eastern populations often shows stronger familial clustering and region-specific molecular patterns, so pedigree analysis, family history collection and targeted testing are relatively effective (40, 41). Besides, there will be considerable genetic differences in the general areas. Founder mutations, linguistic subdivisions and local population histories can affect the regional molecular epidemiology of East Asia as well (42). Therefore, the first goal of panel design should not be to increase the quantity of test items, but rather to enhance the coverage of local diseases in the screening content. In terms of public health, a local panel can improve the diagnostic efficiency and interpretability for people with specific founder mutations or genetically underrepresented groups (Table 5).

**Table 5:** Regional and ethnic diversity in genetic hearing loss and implications for screening strategies.

Population or region	Genetic epidemiological features	Implications for screening and public health
Chinese populations, including eastern and southern China	Commonly implicated genes include GJB2, SLC26A4, and mt-RNR1. In some regions, SLC26A4-related hearing loss associated with enlarged vestibular aqueduct is relatively frequent.	Region-adapted genetic panels may improve diagnostic yield. Early imaging evaluation for suspected enlarged vestibular aqueduct and education on avoiding head trauma may also be useful.
Middle Eastern and Palestinian populations	Higher rates of consanguinity may increase familial aggregation of autosomal recessive hearing loss.	Screening strategies should emphasize detailed family history, pedigree analysis, genetic counseling, and targeted molecular testing when familial inheritance is suspected.
Sub-Saharan African populations	The genetic architecture of hearing loss is highly diverse, and currently known high-frequency variants may explain only a limited proportion of cases.	Direct application of Western or East Asian genetic panels may have limited clinical utility. Locally informed sequencing data are needed to redesign panels and improve clinical interpretation.
Specific East Asian subgroups or founder populations	Founder variants or population-specific variant enrichment may occur, leading to regional differences in variant frequency and diagnostic yield.	Localized screening panels may improve cost-effectiveness, diagnostic yield, and clinical interpretability, especially when supported by population-specific genetic data.

### 3.3 Genotype–phenotype discordance and long-term risk

Genotype–phenotype associations in the context of new molecular technologies for disease research are often more complex than expected. The reasons for incomplete penetrance, variable expressivity and age-dependent progression in the course of hereditary hearing loss in children cannot be ignored (43). It is more serious for newborns and infants, as some pathogenic mutations may be present at birth but do not cause obvious hearing loss. Therefore, there will be a delay between the molecular results and the screening results.

A typical case is the GJB2 p. V37I variant in East Asian populations that is closely linked to mild-to-moderate, delayed-onset, or progressive hearing loss (31). Studies show that hearing loss associated with the biallelic GJB2 p. V37I mutation may be progressively worse in childhood and adolescence. Affected children may have normal or near-normal hearing thresholds at birth but develop clinically recognizable SNHL later (30). In light of this, UNHS will be unable to identify the risk in time. Therefore, the purpose of parallel genetic tests is to find children with serious congenital hearing loss early, as well as those who may develop other health problems in the future (7, 44).

Similar genotype–phenotype discordance is observed in other non-syndromic deafness genes. Certain GJB2 variants, some SLC26A4-related phenotypes, and other low-penetrance pathogenic loci may present with mild onset, fluctuating expression, or progression influenced by environmental triggers (27, 31, 43). Therefore, genetic information in pediatric hearing care should be interpreted primarily as a marker of future risk rather than as a fixed binary diagnosis. In children who carry pathogenic or likely pathogenic variants but have normal hearing at the time of testing, the goal should not be immediate disease labeling, but individualized surveillance, tailored follow-up intervals, and parental education regarding auditory behavior, language milestones, and possible triggers.

This issue highlights the importance of integrating genetic findings with age, audiological phenotype, and longitudinal follow-up. Ignoring the temporal gap between genotype and phenotype may results in two opposing errors: overmedicalization of molecularly positive but clinically asymptomatic children, or false reassurance following a normal newborn hearing screen. Therefore, the most appropriate approach involves using genetic testing as part of a predictive management strategy, particularly when pathogenicity is well established and the natural history of the condition is sufficiently understood.

### 3.4 Complex phenotype: EVA and ANSD

Among hereditary childhood hearing disorders, EVA and ANSD represent two particularly informative complex phenotypes. Both are strongly associated with identifiable molecular etiologies. Nevertheless, they may be inadequately captured by standard newborn hearing screening. In addition, their natural history, audiological profiles, and rehabilitation requirements differ substantially from those of more typical stable cochlear hearing loss.

EVA is one of the most common inner ear abnormalities identified in pediatric imaging and is most frequently associated with SLC26A4 variants, although it may occur in other etiological contexts as well (36). Clinically, EVA is characterized by variable severity, unilateral or bilateral involvement, fluctuating or progressive course, and episodes of sudden deterioration following minor head trauma, pressure change, or infection. Therefore, its diagnostic value is considered both retrospective and predictive: it helps explain existing hearing loss while also identifying future risk of progression. In children suspected of EVA, imaging provides important complementary information alongside molecular testing. Once diagnosed, management should include counseling on activity precautions, injury prevention, and long-term monitoring of auditory trajectory (36).

ANSD represents a phenotypically diverse group of disorders caused by impaired synaptic transmission or neural synchrony within the auditory pathway. Its molecular basis is heterogeneous and includes OTOF, OPA1, and other genes (19, 31, 45, 46). Hereditary ANSD differs from acquired forms related to NICU exposure, hyperbilirubinemia, hypoxia, or other perinatal insults, making etiological stratification critical for prognosis outcomes and treatment planning. A major challenge in ANSD is that pure-tone thresholds often underestimate real-world communication difficulty. Some children may respond relatively well in quiet environments yet have marked impairment in speech understanding under complex listening conditions. As a result, threshold-based severity classification alone is insufficient. Clinical assessment should include ABR, OAE, speech perception, hearing aid benefit, and candidacy for cochlear implantation, while molecular diagnosis can further refine prognostic estimation and therapeutic decision-making (31, 45).

Together, EVA and ANSD illustrate the heterogeneity of hereditary hearing loss regarding lesion site, disease progression, and treatment response. EVA primarily reflects structural inner ear vulnerability, whereas ANSD reflects dysfunction at the level of the synapse or auditory nerve. Thus, accurate classification and individualized care require molecular findings to be interpreted in conjunction with imaging, audiological phenotype, and functional outcomes.

### 3.5 Recognizing warning signs of syndromic hearing loss

Although non-syndromic hearing loss accounts for most hereditary pediatric hearing impairment, recognition of syndromic hearing loss remains clinically essential. Its significance lies not in frequency. Rather, it lies in the fact that some syndromes involve multisystem disease, which may be life-threatening or strongly influence long-term prognosis, with hearing loss serving as an early and accessible clinical clue.

A classic example is Jervell and Lange-Nielsen syndrome, which combines profound congenital sensorineural hearing loss with long QT syndrome and carries a high risk of malignant arrhythmia and sudden death (47). In children with bilateral severe congenital hearing loss of unknown etiology, routine electrocardiographic evaluation is particularly important, as early identification of cardiac conduction abnormalities may be lifesaving. In other disorders, such as osteogenesis imperfecta, hearing loss may occur alongside abnormal bone fragility, reminding clinicians that audiological findings may be one component of a broader multisystem genetic disease (48).

A further diagnostic challenge is that syndromic hearing loss may initially present as isolated auditory impairment, with extra-auditory manifestations appearing only later in childhood. Therefore, clinicians should remain alert to signals such as hearing loss accompanied by arrhythmia, visual symptoms, vestibular dysfunction, skeletal abnormalities, thyroid disease, renal anomalies, developmental delay, or strong familial clustering. When such features are present, early multidisciplinary referral is necessary and may include genetic counseling, cardiac assessment, imaging, and other specialty evaluations as indicated. From a public health perspective, the value of identifying syndromic hearing loss lies in the possibility of improving outcomes beyond hearing itself through timely recognition of a small but high-risk subgroup (Table 6).

**Table 6:** Early warning signs of complex phenotypes and multisystem involvement.

Clinical category	Key clinical features and potential risks	Clinical management and response strategies
Genotype–phenotype discrepancy, such as GJB2 p. V37I	Some variants may show incomplete penetrance or variable expressivity. Auditory function may be normal or near-normal at birth, whereas hearing loss may emerge or progress with age.	A molecular-positive result should be interpreted as a risk-stratification marker rather than as an immediate indication of disease. Long-term individualized monitoring is recommended to avoid both delayed diagnosis and overmedicalization.
EVA-associated phenotype	Hearing loss may be fluctuating or progressive. Deterioration can occur after minor head trauma, barometric pressure changes, or infection. Imaging may reveal enlarged vestibular aqueduct or related inner-ear abnormalities.	Genetic findings should be interpreted together with imaging results. Lifestyle counseling, avoidance of head trauma, follow-up audiological monitoring, and prognostic assessment are important.
ANSD-associated phenotype	Pure-tone thresholds may not fully reflect real-world communication difficulty. Speech perception can be disproportionately impaired, especially in complex listening environments.	Assessment should not rely only on hearing-threshold measures. Speech recognition, functional auditory performance, hearing-aid benefit, and candidacy for cochlear implantation should be evaluated when appropriate.
Indicators of syndromic hearing loss, such as Jervell and Lange-Nielsen syndrome	Hearing loss may coexist with long QT syndrome, which is associated with malignant arrhythmia and sudden death. Other warning signs may include visual, skeletal, renal, cardiac, or developmental abnormalities.	Hearing abnormalities may serve as early clues to syndromic disease. When multisystem warning signs are present, multidisciplinary evaluation should be initiated promptly, including electrocardiography and relevant specialty assessments.

In summary, the hallmark of hereditary childhood hearing loss is profound heterogeneity. Differences in genes, variants, and population background shape not only the occurrence of hearing loss, but also its age at onset, rate of progression, pathological substrate, and opportunities for intervention. Genetic etiological research is important not simply because it improves diagnostic yield, but because it supports the transition of pediatric hearing care from an empirical model toward one based on molecular stratification, regional adaptation, and precision follow-up.

## 4 Infectious etiologies

### 4.1 Congenital cytomegalovirus infection and its role in delayed-onset hearing loss

cCMV is one of the leading non-genetic causes of childhood SNHL and has become increasingly important in the UNHS era (13, 49, 50). In contrast to many congenital disorders identifiable at birth, cCMV is clinically challenging owing to its often asymptomatic presentation. Most maternal and neonatal infections are not accompanied by obvious symptoms at delivery, and affected infants may pass newborn hearing screening despite being at risk of developing delayed-onset, progressive, or fluctuating SNHL during infancy or later childhood (51–53). cCMV therefore represents one of the main explanations for children who initially pass screening but later develop hearing loss (Figure 2).

**Figure 2:**
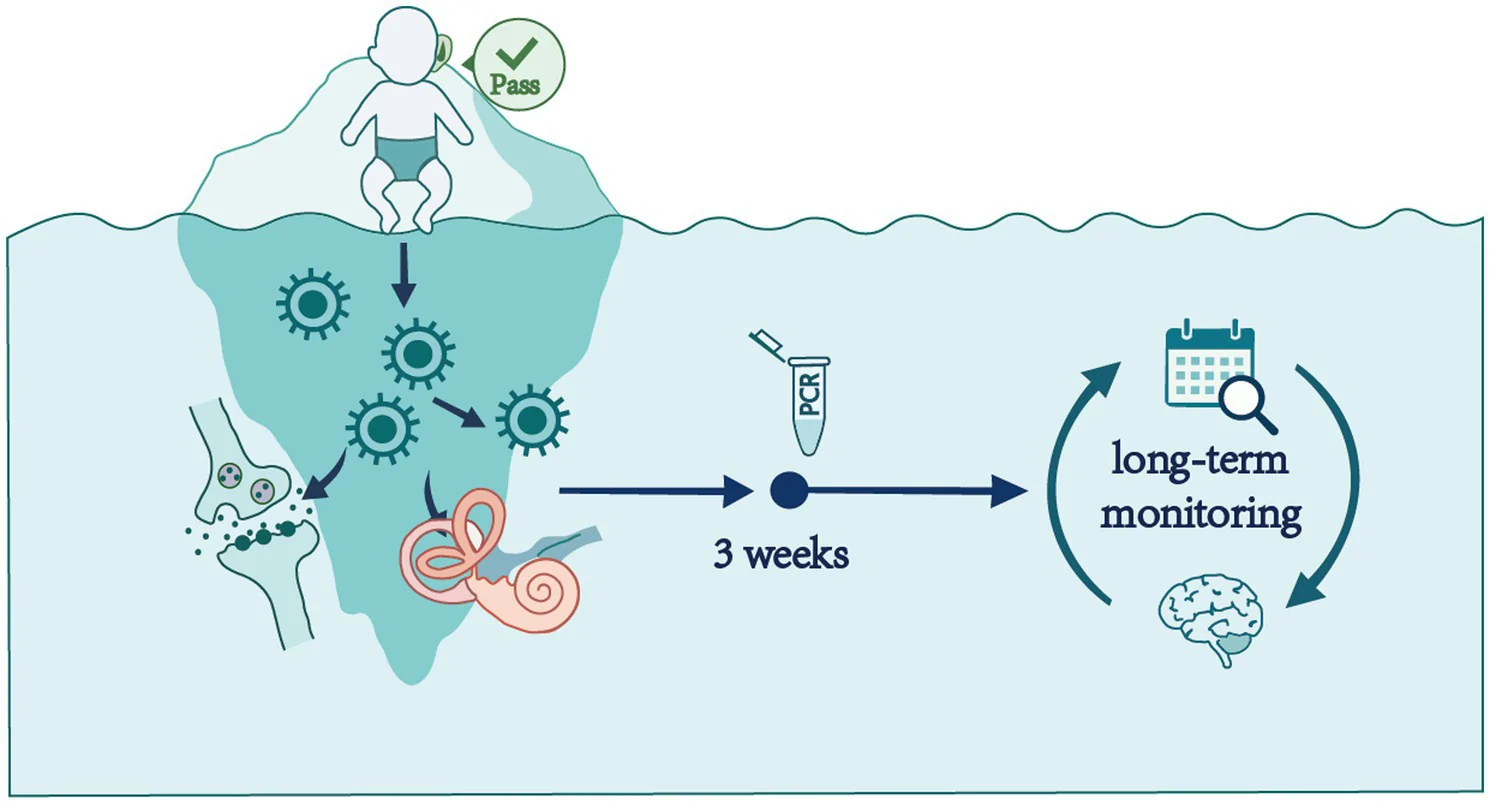
Congenital cytomegalovirus infection and delayed-onset pediatric hearing loss.

The auditory phenotype of cCMV is highly variable. Hearing loss may be unilateral or bilateral, stable or fluctuating, and may worsen over time, with progression differing between ears and across various developmental stages (49, 54). Proposed mechanisms include direct viral injury to inner ear structures, persistent inflammatory responses, immune-mediated damage, and involvement of the central nervous system (55). Unlike more stable forms of congenital hearing loss, cCMV-related auditory impairment often follows a non-linear course. Some children have normal hearing at birth and subsequently develop mid- or high-frequency loss, unilateral progression, or bilateral deterioration. Consequently, cCMV should be regarded not simply as a perinatal infection, but as an early-life exposure associated with sustained neuro-otological risk.

For this reason, effective prevention and management require integration of pathogen testing into newborn screening and early hearing detection and intervention (EHDI) frameworks. Saliva or urine polymerase chain reaction (PCR) testing is currently the main diagnostic approach, with important value in identifying infants during the early postnatal period before overt hearing or neurological abnormalities become apparent (56, 57). Accurate diagnosis depends on strict timing: testing should ideally be completed within the first 3 weeks of life to distinguish congenital from postnatal infection (58). Beyond this window, standard virological testing becomes much less informative for attributing later hearing loss to congenital infection.

A confirmed diagnosis of cCMV provides information about long-term auditory risk, particularly because of the fact that hearing loss may be delayed, progressive, unilateral, bilateral, or fluctuating. Imaging abnormalities, viral burden, central nervous system involvement, and clinical manifestations at birth may all influence long-term auditory and neurodevelopmental outcomes (13, 54, 55, 58). Monitoring should therefore extend beyond the neonatal period and include repeated hearing assessment throughout infancy and early childhood, with attention to threshold fluctuation, laterality, speech-language development, and broader neurological maturation. In selected symptomatic infants, antiviral therapy may improve long-term outcomes, particularly by supporting hearing stability and facilitating earlier intervention (59). From both clinical and public health perspectives, cCMV illustrates why etiological information is important for interpreting auditory risk after a normal newborn hearing screen (60).

### 4.2 Assessing the evidence on prenatal SARS-CoV-2 exposure and neonatal auditory outcomes

The possible effect of maternal severe acute respiratory syndrome coronavirus 2 (SARS-CoV-2) infection on auditory outcomes in offspring has attracted attention in both maternal-fetal medicine and pediatric audiology (61, 62). This concern is biologically plausible, as viral infection during pregnancy may influence fetal development through placental inflammation, maternal immune activation, fetal hypoxia, or neonatal complications. However, current evidence does not support a consistent causal association between prenatal SARS-CoV-2 exposure and persistent childhood hearing loss.

To date, most retrospective cohorts, multicenter observational studies, and systematic reviews have not uncovered a robust or reproducible relationship between maternal SARS-CoV-2 infection and abnormal newborn hearing screening results or permanent hearing impairment in offspring (63–65). Some reports have described transient screening abnormalities, slightly lower initial pass rates, or isolated auditory findings, but these observations often do not persist on rescreening or longer follow-up and are vulnerable to confounding effects by prematurity, NICU admission, prenatal care disruption, and limited sample size (64, 65). At present, SARS-CoV-2 is better considered an emerging exposure warranting continued observation rather than a definite high-risk factor for childhood hearing loss.

More broadly, infectious causes differ markedly in the strength of evidence, feasibility of intervention, and public health relevance. Within this spectrum, cCMV has clear etiological significance and actionable implications, whereas the evidence for SARS-CoV-2 remains preliminary. Existing data are insufficient to support routine etiological screening or specialized auditory surveillance based solely on maternal SARS-CoV-2 exposure during pregnancy. In resource-constrained systems, screening and follow-up efforts should remain focused on conditions with stronger evidence, clearer disease burden, and more practical intervention pathways. Future clarification will depend on well-designed birth cohorts with long-term follow-up and better management of confounding factors, particularly for combined auditory and neurodevelopmental outcomes.

### 4.3 Other infection-related factors: congenital syphilis, HIV-exposed uninfected children, and postnatal infectious burden

In addition to congenital CMV, several other infections and infectious causes of hearing loss in childhood are possible, and their mechanisms and management requirements are significantly different. Some functions are relatively discrete etiological triggers that require prolonged observation, while others are broad composite risk states caused by adverse perinatal and postnatal conditions.

Congenital syphilis is a preventable infectious cause of hearing loss in children that needs to be tackled. Although the prevalence varies among areas according to the public health management measures in those areas, both clinical and pathological evidence support an association with delayed-onset and progressive hearing loss (66). Hearing loss due to syphilis in infants and young children may occur at any time, and the degree of damage is also irregular. Therefore, children with documented congenital syphilis or confirmed prenatal exposure should be subject to extended follow-up even if the newborn hearing screening results are normal. Therefore, the history of illness will be more helpful than a single normal screening result at birth.

HIV-exposed uninfected (HEU) children represent another population of interest. In this group, hearing risk is typically not attributable to a single pathogen-specific mechanism, but rather to the cumulative effects of maternal illness, in utero drug exposure, prematurity, low birth weight, perinatal complications, and increased susceptibility to postnatal infections (67). HEU children are therefore better understood as part of a broader infection-related composite risk cluster than as a classic post-infectious hearing loss phenotype. Their auditory follow-up should be integrated with surveillance for other perinatal vulnerabilities and should include neurodevelopment, infection history, NICU exposure, and language outcomes, rather than focusing on HIV exposure in isolation.

Postnatal infectious burden is also an important contributor to childhood hearing loss, particularly in resource-constrained settings. Chronic suppurative otitis media (CSOM) and other recurrent middle ear infections are major causes of acquired hearing loss in children (68). Although these disorders typically produce conductive loss, prolonged inflammation may also damage inner ear structures or worsen pre-existing vulnerability. Their public health importance extends beyond infection itself, as risk is closely linked to sanitation, healthcare access, delayed treatment, and inadequate home care resources. In this sense, infection-related hearing loss is also a problem of health inequity. For these children, hearing protection depends as much on early detection and systematic infection management as on audiological services.

Taken together, infection-related childhood hearing loss is not a single category dominated by one pathogen, but a heterogeneous group of conditions influned by pathogen type, timing of exposure, host susceptibility, and broader prenatal or postnatal context. cCMV remains the best-established and most actionable infectious etiology; congenital syphilis illustrates the need for etiology-based long-term follow-up; HEU children highlight the contribution of cumulative perinatal risk; and chronic postnatal infections show that hearing loss prevention must be linked closely to infection management and primary care systems. Consequently, management should move beyond pathogen recognition alone toward risk-adapted strategies for testing, follow-up, and intervention.

## 5 Intrauterine environment and modifiable exposures

### 5.1 Maternal nutritional status and auditory neural development: iron deficiency and anemia

Normal fetal auditory development requires good nutritional support in the womb, and maternal iron status is a typical example. Iron is needed for the production of haemoglobin and oxygen transport, as well as the development of oligodendrocytes, myelination, mitochondrial metabolism, and neurotransmitter synthesis. As a result, maternal iron deficiency and iron-deficiency anaemia (IDA) may harm the development of the fetal brainstem auditory pathway, reduce the efficiency of synaptic transmission, interfere with neural plasticity, and thus increase the risk of hearing impairment later in life (69, 70). Unlike structural malformations, the effects of such exposure may manifest as functional developmental abnormalities rather than overt anatomical defects. Clinically, these problems may not be shown as raised hearing thresholds in early life but rather as prolonged ABR latencies, impaired temporal processing, or later deficits in auditory processing and language development.

From a developmental perspective, the mid-to-late gestation period is critical for fetal myelination and the maturation of the auditory pathway. Chronic iron deficiency during this timeframe can compromise neural conduction and brainstem integration, thus altering early postnatal auditory responses. Maternal–infant cohort studies have indicated an association between neonatal iron deficiency and latent auditory impairment in infancy, although this relationship may also be influenced by socioeconomic and healthcare factors (69). This finding has significant public health implications: iron deficiency in pregnancy is prevalent, preventable, and strongly associated with structural disadvantages, including poverty, malnutrition, and inadequate prenatal care. It may therefore act as an upstream determinant of hearing health inequity in children.

From a preventive standpoint, managing maternal iron levels should be integrated into primary prevention strategies for childhood hearing loss. Routine antenatal screening for anemia, alongside appropriate iron supplementation, can enhance the fetal neurodevelopmental environment while reducing adverse perinatal outcomes. Compared to postnatal interventions, optimizing maternal nutrition during pregnancy provides a critical time advantage by minimizing risk before developmental injury occurs. Hence, maternal nutrition should be incorporated into etiological frameworks for childhood hearing loss not merely as background context but as a potentially modifiable exposure with direct preventive relevance (71).

### 5.2 Endocrine and metabolic milieu: thyroid dysfunction and gestational metabolic disorders

In addition to nutrition, maternal endocrine and metabolic conditions significantly influence the formation of auditory structures and the maturation of neural pathways. Thyroid hormones are essential for cochlear development, hair cell differentiation, ion transport regulation, and auditory nerve myelination. During early and mid-gestation, the fetal nervous system relies heavily on maternal thyroid hormone supply. Untreated maternal hypothyroidism or insufficient thyroid hormone availability during this period may impair fetal auditory development and increase the risk of later SNHL (72–74). Unlike infectious or genetic causes, thyroid dysfunction rarely generates a distinctive audiological phenotype, which may contribute to its frequent under-recognition in etiological assessments despite its high prevalence and clear potential for intervention.

Maternal metabolic disorders, particularly gestational diabetes mellitus (GDM) and chronic hyperglycemia, may also affect fetal auditory development through various mechanisms. Hyperglycemia can disrupt placental and fetal microcirculation, elevate oxidative stress, and promote chronic low-grade inflammation, thereby impacting the cochlear microenvironment and neural tissue homeostasis (75, 76). Given that the inner ear relies on a specialized vascular supply, prolonged exposure to an adverse metabolic environment may heighten the vulnerability of both sensory and supporting structures. Additionally, maternal metabolic disturbances may interact with fetal genetic susceptibility, amplifying risk in specific individuals (76). This highlights the notion that childhood hearing loss often arises from interactions between biological susceptibility and the developmental environment rather than from a single, isolated cause.

Immune-metabolic dysregulation may also be involved. Maternal autoimmune disease, abnormal inflammatory activation, and immune dysregulation in pregnancy can affect the development of fetal inner ears and the nervous system through placental inflammation, thus leading to silent auditory defects (77). Although evidence at the population level is still lacking, the above mechanisms suggest that metabolic and immune homeostasis are necessary conditions for the intrauterine auditory environment. Collectively, thyroid dysfunction, abnormal glucose metabolism, and other immune-metabolic disorders should be considered modifiable prenatal risk factors and receive more attention in etiological investigation.

### 5.3 Environmental and lifestyle exposures: tobacco, chemicals, drugs, and noise

The intrauterine auditory environment is shaped not only by maternal disease and nutrition but also by lifestyle behaviors and environmental exposures during pregnancy. Among these factors, tobacco and nicotine exposure are the most well-documented and relevant from a public health perspective. Nicotine and related toxic compounds have the ability to reduce placental perfusion, promote chronic fetal hypoxia, and exert direct neurotoxic effects on the developing nervous system (78, 79). Active smoking, secondhand smoke exposure, and potentially exposure to newer nicotine products have all been associated with increased risk of auditory impairment in offspring, with some evidence of dose dependence. Smoking cessation during pregnancy and avoidance of passive smoke exposure should therefore be considered basic primary prevention measures for childhood hearing loss.

Environmental toxicants and heavy metals are now known to be the source of risk. Developmental toxicology and environmental epidemiology studies have shown that organophosphate pesticides, persistent organic pollutants, bisphenol compounds, and heavy metals such as lead can interfere with the development of fetal sensory organs and neural maturation, potentially leading to later auditory dysfunction (80–82). A long-standing problem in this body of research is that the exposure often involves mixed low-dose effects, is difficult to quantify accurately, and is subject to socioeconomic and residential disparities. Thus, the population studies may yield different results. Nevertheless, their neurotoxicity and wide dispersion in the environment also warrant their inclusion in the scope of etiology for polluted areas and exposed populations.

Medication exposure during pregnancy also requires careful evaluation. Some medications may negatively impact fetal auditory development in addition to imparting general developmental risks (83–85). Recent reviews suggest a possible association between certain maternal drug exposures and congenital or early-onset hearing loss in children, although the strength of evidence varies widely by drug class and is often confounded by underlying maternal conditions, co-medication use, and pregnancy complications (83). For this reason, pharmacological risk assessments should remain contextual, avoiding both under-recognition of possible ototoxicity and overly simplistic causal inferences. Decisions regarding potentially relevant medications should involve careful risk–benefit evaluations, considering gestational timing, disease severity, and alternative treatment options.

Evidence regarding prenatal noise exposure remains limited and is largely derived from animal studies and mechanistic inferences. Experimental data show that prolonged exposure of the mother to high-intensity noise can affect the development of fetal hearing through activation of the stress axis, glucocorticoid dysregulation, and potentially mechanical transmission pathways, resulting in altered hearing thresholds or synaptic damage in the offspring (86). Although there is no direct evidence, some research has been conducted to explore whether non-chemical environmental factors in utero are also related to increased auditory risks. Generally speaking, environmental and lifestyle exposures are relatively easy to modify and thus suitable targets for primary prevention of childhood hearing loss. Genetic factors and some infectious diseases are not usually due to such exposures; others can be prevented by health education, occupational health, prenatal care and broad policies. The above factors should be included in the etiology system and are likely to be affected by society and, thus, may be altered (Table 7).

**Table 7:** Prenatal environmental exposures and potential mechanisms of impaired auditory development.

Exposure category	Major risk factors	Potential mechanisms affecting auditory development	Clinical or audiological phenotype
Nutritional status	Maternal iron deficiency or iron-deficiency anemia.	Iron deficiency may impair oligodendrocyte differentiation, myelin formation, brainstem maturation, and auditory pathway integration.	Some children may show hidden functional impairment, including prolonged auditory brainstem response latency, impaired temporal processing, and subsequent language-processing difficulties, even when hearing thresholds appear normal.
Endocrine and metabolic conditions	Maternal hypothyroidism or inadequate thyroid hormone availability.	Thyroid hormone deficiency may affect inner-ear development, hair-cell differentiation, ion-transport homeostasis, and auditory nerve myelination.	There may be an increased risk of sensorineural hearing loss. Clinical phenotypes are often nonspecific and may therefore be underestimated.
Endocrine and metabolic conditions	Gestational diabetes mellitus or persistent maternal hyperglycemia.	Hyperglycemia may contribute to microcirculatory dysfunction, oxidative stress, and increased vulnerability of cochlear sensory cells and supporting structures.	A prolonged adverse metabolic environment may interact with genetic susceptibility and increase the risk of auditory developmental impairment.
Lifestyle-related exposures	Active or passive tobacco and nicotine exposure, including novel tobacco products.	Nicotine and related exposures may induce placental vasoconstriction, chronic fetal hypoxia, and neurodevelopmental toxicity.	Prenatal tobacco or nicotine exposure has been associated with an increased risk of childhood auditory impairment, with possible dose-dependent effects.
Environmental chemical exposures	Pesticides, bisphenol compounds, and heavy metals such as lead.	These exposures may interfere with sensory-organ maturation and nervous-system development and may exert neurotoxic effects.	Childhood auditory impairment may be influenced by combined environmental, socioeconomic, and household exposure factors.
Medication and physical exposures	Potentially ototoxic medications during pregnancy and exposure to high-intensity noise.	Medications may exert developmental neurotoxic or ototoxic effects. Noise exposure may activate the maternal-fetal stress axis, alter glucocorticoid regulation, or affect auditory transmission.	These exposures may increase the risk of congenital or early-onset auditory dysfunction. Clinical interpretation should consider exposure timing, dose, duration, and the maternal-fetal risk–benefit balance.

### 5.4 Paternal influences and epigenetic signals

Historically, research on the intrauterine environment has primarily focused on maternal variables. Recent data suggest that paternal influences may also impact embryonic and fetal development through epigenetic pathways, subsequently affecting the long-term health of the offspring’s sensory systems. Potential mechanisms of paternal exposure include altered sperm DNA methylation, irregular histone modifications, and variations in non-coding RNA expression. These molecular changes can influence early embryonic development post-fertilization and regulate organogenesis and the maturation of the nervous system (87). Although direct population evidence linking paternal factors to offspring auditory development is limited, transgenerational developmental biology research indicates that unhealthy lifestyles, toxic exposures, and chronic metabolic disorders in fathers may affect neurobehavioral and sensory system phenotypes in offspring through epigenetic reprogramming of germ cells.

Research on childhood hearing loss has pointed out the influence of fathers at two levels. Harmful parenting behavior is also a relatively unknown risk factor for development. Chronic alcohol consumption, exposure to environmental pollutants and other adverse factors may alter the susceptibility of offspring to neurotoxicity and metabolic disorders through epigenetic changes in sperm (87, 88). Fathers’ factors are also associated with genetic counseling and the identification of family risks. When both parents are deaf or if there is a cluster of deafness-causing genes in the family, the paternal line has a distinct history of genetic diseases and therefore requires an assessment of the child’s hearing risk that extends beyond the mother’s medical history to include an all-encompassing view of the family (89).

Currently, paternal factors mainly reside in the realm of preliminary investigation and do not yet warrant the establishment of standardized clinical monitoring protocols comparable to those for maternal illnesses, dietary deficiencies, or NICU exposure. However, their academic significance lies in shifting the etiology of childhood hearing loss from a solely maternal-focused framework to a more inclusive model that takes into account reproductive and developmental exposures. If future prospective cohort studies can validate the independent impacts of paternal lifestyle and environmental exposures on offspring auditory outcomes, prenatal care and primary prevention programs must expand from maternal interventions to include joint parental strategies. Integrating paternal variables as epigenetic indicators into contemporary discussions provides a more holistic understanding of how intrauterine and periconceptional exposures affect the development of the auditory system in infants (Table 8).

**Table 8:** Advancements in Paternal Influence and Epigenetics Investigation.

Assessment criteria	Fundamental academic viewpoints and possible mechanisms	Clinical and research importance
Epigenetic pathways	Paternal exposure to adverse lifestyle factors or environmental chemicals may alter sperm epigenetic profiles, including DNA methylation, histone modifications, and small non-coding RNAs. These changes may influence early embryonic development and subsequent neurodevelopmental programming.	This perspective suggests that paternal exposures may contribute to offspring brain and sensory-system phenotypes through germline-mediated developmental regulation.
Expansion of genetic counseling	Risk assessment should not be limited to maternal factors. Paternal medical history, occupational or environmental exposures, lifestyle factors, and familial genetic predispositions should also be considered.	This supports a shift from a primarily “maternal-centered” etiological framework to a broader “parental reproductive and developmental exposure model.”
Improvement of preventive strategies	If future prospective cohort studies confirm an independent paternal effect, preventive interventions should be extended from the prenatal period to the preconception stage and involve both parents.	Although this field has not yet formed routine clinical pathways, it provides an important theoretical basis for early prevention and more comprehensive developmental-risk assessment.

Intrauterine environmental risk factors are a highly preventable cause of childhood hearing loss. Unlike genetic factors, nutritional deficiencies, endocrine and metabolic issues, exposure to tobacco and environmental toxins, certain medications, and stressors present distinct avenues for intervention. A shared characteristic among these factors is that they do not consistently produce observable elevated hearing thresholds at birth; instead, they contribute to delayed or masked auditory functional impairments by influencing auditory nerve maturation, inner ear homeostasis, and various developmental processes. Therefore, managing the intrauterine environment in the prevention and management of childhood hearing loss should be integrated into standard prenatal care and recognized as a primary prevention strategy aimed at enhancing long-term auditory and neurological developmental outcomes.

## 6 Perinatal and NICU risks

### 6.1 Preterm birth and underdeveloped auditory susceptibility

Perinatal and NICU-associated hazards represent a substantial factor in non-genetic hearing loss among children, with preterm birth and low birth weight serving as the primary biological vulnerabilities. Compared to full-term infants, premature infants—particularly those with extremely low birth weight—exhibit underdeveloped cochlear hair cells, impaired microcirculation of the stria vascularis, inadequate myelination of the auditory nerve, and compromised brainstem auditory pathways. Consequently, they demonstrate an increased vulnerability to hypoxia, inflammation, metabolic abnormalities, and pharmacological exposure (14). These vulnerabilities may not manifest as immediate hearing threshold anomalies at birth; instead, they significantly raise the likelihood of later SNHL, ANSD, and compromised central auditory function.

From a developmental perspective, the auditory system undergoes substantial growth during the perinatal phase. Factors that affect oxygen delivery, blood perfusion, and energy metabolism equilibrium can have long-lasting effects on the inner ear and central auditory pathways. Metabolomics and related studies indicate that premature newborns exposed to perinatal hypoxia, acidosis, or systemic inflammatory responses are at heightened risk for disturbances in metabolic homeostasis within the inner ear and cerebral tissues. This increases the likelihood of sensory cell damage, disruption of synaptic transmission, and delayed maturation of neural circuits (18, 90). Additionally, premature newborns may experience complications such as middle ear effusion, prolonged intubation, and issues related to mechanical ventilation, which further heightens their susceptibility to both sensorineural and conductive hearing loss (18). As such, in perinatal risk assessment, preterm birth should be considered more than just a generic background variable; it is a pivotal developmental exposure that influences subsequent hearing monitoring measures. A normal hearing screening result prior to discharge does not definitively rule out later auditory vulnerability in preterm or very-low-birth-weight infants. Effective management must depend on early identification of risk factors, incorporated into a continuous postnatal follow-up protocol that adaptively modifies follow-up frequency based on gestational age, birth weight, intensity of key exposures, and neurodevelopmental outcomes. Therefore, preterm delivery and developmental immaturity not only establish the biological foundation for the heightened risk of hearing loss in the NICU population but also serve as critical stratification criteria within the perinatal hearing screening and management framework.

### 6.2 Ototoxicity induced by pharmaceuticals and the dual-strike mechanism

Ototoxicity of drugs used in the NICU is a relatively serious cause of hearing loss for children. The reasons for this are multifaceted, and they can all be traced back to the lack of development. The initial injury is caused by the damaged state of the inner ear and auditory nerve due to reasons such as immaturity, hypoxia, infection, unstable blood supply and metabolic disorders. The second kind of damage caused by ototoxic drugs includes aminoglycoside antibiotics, loop diuretics and some critical care drugs. Therefore, the standard drug dose may cause more serious and prolonged hearing loss in NICU infants (Figure 3).

**Figure 3:**
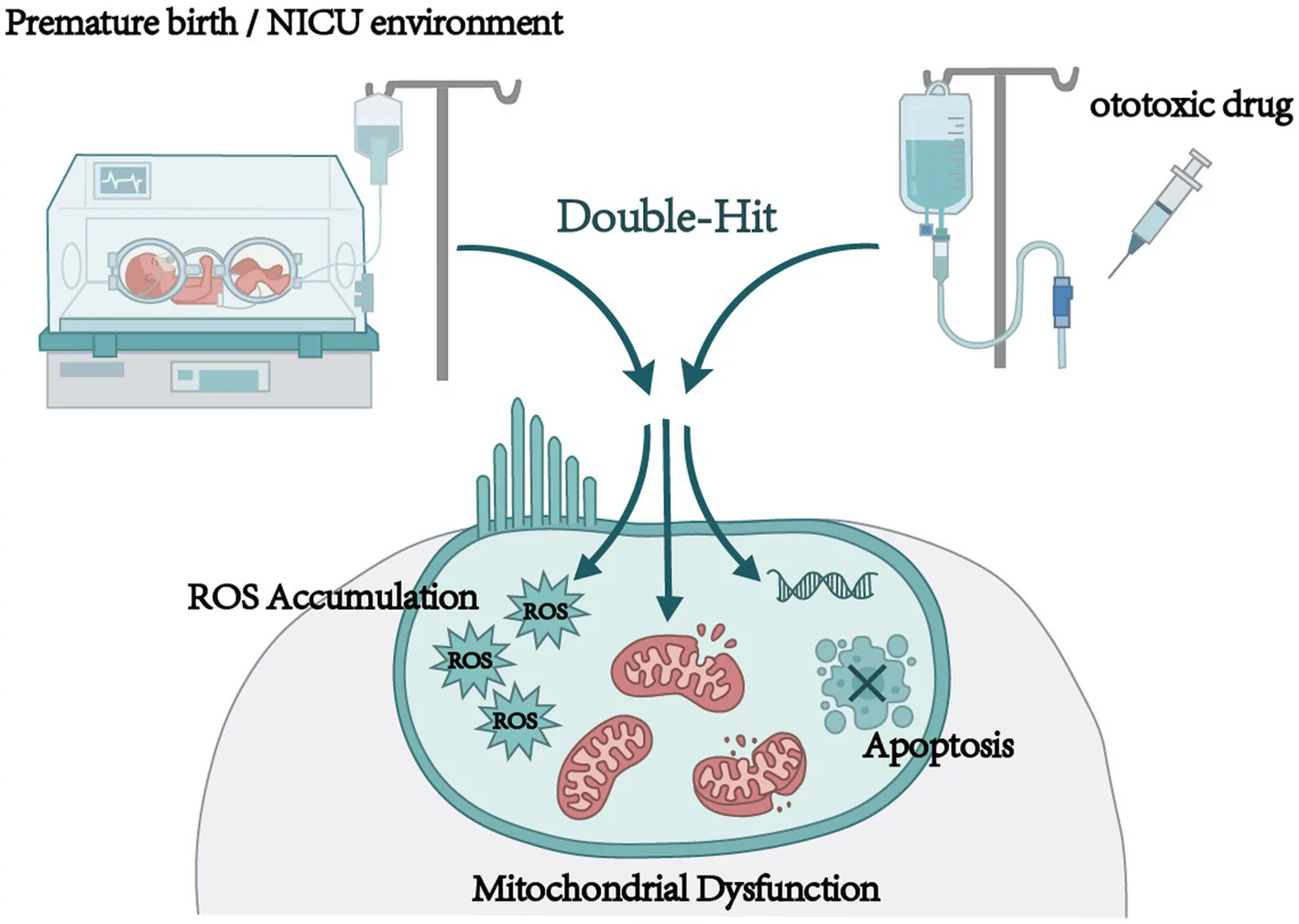
Drug-induced ototoxicity and auditory vulnerability in the neonatal intensive care unit environment.

The main pathways of drug-induced ototoxic damage include the accumulation of reactive oxygen species, mitochondrial dysfunction, death of hair cells, and disruption of microcirculation in the stria vascularis (91, 92). While the research on cisplatin-induced ototoxicity primarily originates from pediatric oncology, the mechanisms of oxidative stress and sensory cell death it reveals significantly enhance our understanding of the molecular underpinnings of common ototoxic exposures in the NICU (93). In the developing inner ear, drug exposure can damage outer hair cells, disrupt synaptic connections, and destabilize neuronal transmission, leading to increased hearing thresholds, compromised speech recognition, or subtler disruptions in neural synchrony.

Genes are also involved in determining susceptibility to ototoxicity induced by drugs. Mutations in the mitochondrial gene mt-RNR1 significantly increase susceptibility to aminoglycoside antibiotics, and exposure to typical therapeutic concentrations may result in permanent hearing impairment. Therefore, pharmaceutical safety management in the NICU needs to consider not only the requirement and time of pharmacotherapy but also personalised risk assessment. In high-risk neonates, multiple ototoxic drugs should be avoided at the same time as much as possible, especially in cases of hypoxia, sepsis or unstable kidney function. Therapeutic Drug Monitoring (TDM) and assessments of genetic susceptibility can be used to reduce the risk of preventable hearing loss.

From a clinical management perspective, the primary strategy for preventing and treating ototoxicity from drugs is not to eliminate essential critical care treatments but to optimize exposure pathways. Audiological follow-up protocols should be established for pediatric patients receiving high-risk medications. Future assessments must focus closely on mild to moderate SNHL, delayed progression, and the nuances of ANSD subtypes. Thus, medication-induced ototoxicity should not be regarded as an isolated adverse effect but should be integrated into the broader NICU multiple exposure framework, where it is assessed alongside developmental immaturity and critical illness to better elucidate the risk of hearing loss.

### 6.3 NICU risk cluster: hyperbilirubinemia, hypoxia, infection, and supportive care

Hearing loss in NICU children generally arises from the combined effects of various significant exposures rather than from a singular cause. Therefore, adopting a risk cluster perspective offers a more accurate representation of the clinical reality than a single-cause approach. These risk clusters commonly include hyperbilirubinemia, prenatal hypoxia, sepsis or severe infection, hemodynamic instability, mechanical ventilation, and exposure to various supportive medications (94–96). The interplay of multiple factors affects the cochlear microenvironment, auditory nerve transmission, and the development of central auditory pathways.

Severe hyperbilirubinemia is a critical neurotoxic risk factor for hearing loss associated with NICU stays. Unconjugated bilirubin is capable of crossing the immature blood–brain barrier, causing preferential damage to the brainstem auditory nuclei and auditory nerve conduction pathways, and is significantly linked to acquired ANSD (19, 20). A major concern with these pediatric patients is that standard newborn hearing screening, particularly when based predominantly on OAE, may inadequately detect neurosynaptic damage, leading to an underestimation of clinical risk. Therefore, for newborns with a history of significant hyperbilirubinemia, subsequent evaluations should emphasize an integrated assessment of both OAE and ABR.

Perinatal hypoxia and prolonged mechanical ventilation primarily impair the cochlea and central nervous system due to inadequate perfusion, oxidative stress, and subsequent inflammatory responses. The vascular network within the inner ear is metabolically active and requires high levels of oxygen, thus making it especially vulnerable to hypoxia and fluctuations in blood flow. Extended exposure to hypoxia or dependency on mechanical ventilation can lead to lasting damage to both the auditory organs and brain networks. Moreover, sepsis and systemic inflammation exacerbate these issues, with effects that transcend acute tissue damage and may increase the risk of later auditory problems due to neurodevelopmental disturbances induced by inflammation.

Additionally, hemodynamic instability and the administration of pressor agents or positive inotropic medications may serve as indirect risk markers. Such exposure often indicates that the patient is experiencing acute circulatory instability and compromised organ perfusion, during which the microcirculation of the inner ear may suffer from ischemia–reperfusion injury (97, 98). While further elucidation of the mechanistic relationship between such drugs and hearing loss is needed, their significance as indicators of severe disease and variations in perfusion is clear. Consequently, the NICU hearing risk assessment should move from a singular event recording paradigm to a systematic exposure integration strategy (Figure 4). This involves the comprehensive integration of factors such as preterm birth, hypoxia, hyperbilirubinemia, infection, ototoxic medications, and the level of supportive care into an electronic risk profile. This profile would then dictate the frequency of post-discharge follow-ups and the necessary combination of tests. For high-risk groups within the NICU, the aim of hearing follow-up should be not only to detect established hearing threshold abnormalities but also to identify potential neuroconductive damage and developmental vulnerabilities, thereby minimizing diagnostic delays.

**Figure 4:**
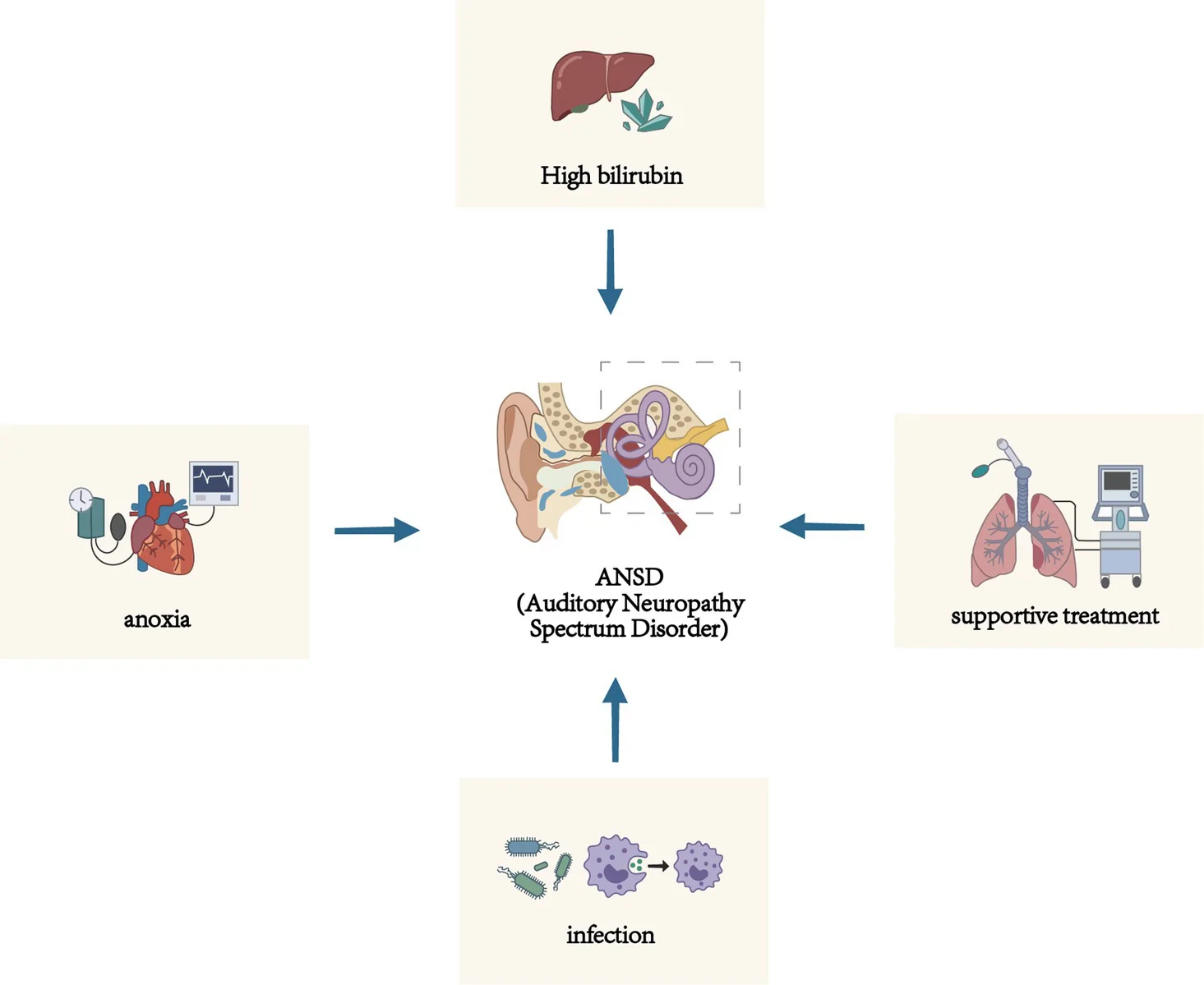
Multifactorial neonatal intensive care unit exposures and auditory pathway vulnerability.

### 6.4 Imaging assessment and causal localization: inner ear anomalies and Cochlear nerve irregularities

Imaging investigations provide substantial benefits in the etiological evaluation of perinatally high-risk children and newborns with congenital severe hearing loss. High-resolution computed tomography (CT) and magnetic resonance imaging (MRI) allow for clear visualization of the anatomical features of the inner ear and can detect cochlear nerve developmental abnormalities, inner ear malformations, and related central structural anomalies. These imaging modalities are essential for diagnosing the etiology, evaluating prognosis, and informing decisions regarding auditory reconstruction (99, 100). Imaging assessment is particularly essential for children who exhibit abnormal results on routine hearing screenings, present inconsistent audiological findings over time, or face specific challenges, such as unilateral profound hearing loss or multiple developmental abnormalities (Figure 5).

**Figure 5:**
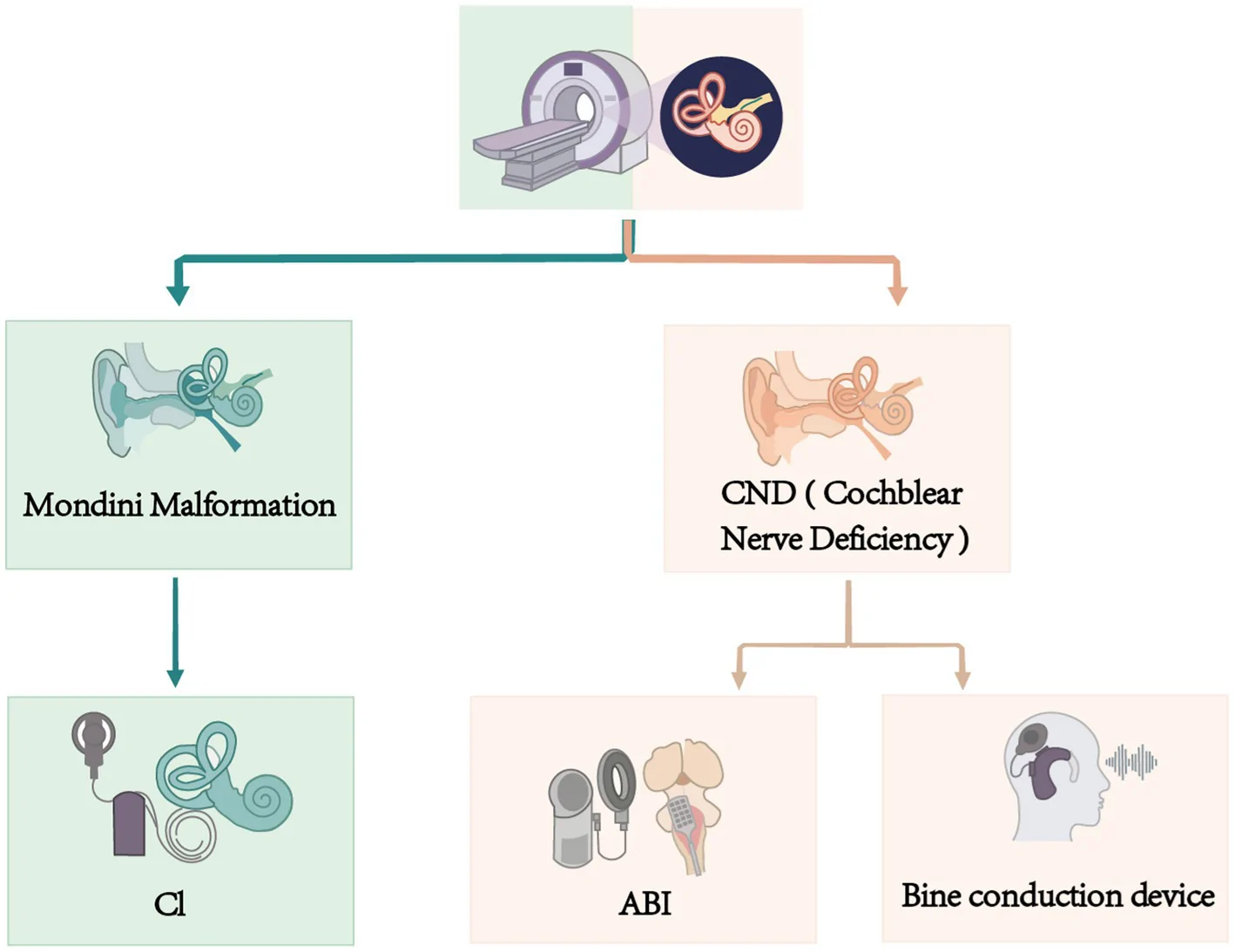
Imaging assessment and anatomical etiologies of pediatric hearing loss.

Congenital malformations of the inner ear, such as Mondini malformation, common cavity malformation, and various cochlear or vestibular developmental abnormalities, are significant causes of severe or profound hearing loss (101). These lesions not only help clarify the origin of the hearing loss but may also indicate clinical risks, including cerebrospinal fluid leaks, recurrent meningitis, or increased complexity during surgical interventions. Thus, imaging assessment in these pediatric patients is not merely an anatomical adjunct; it is essential for establishing therapeutic strategies. Molecular testing can further validate imaging results in children with EVA or other imaging abnormalities, enhancing the accuracy of etiological classification.

Cochlear nerve deficiency (CND) is a primary focus for imaging evaluation in cases of unilateral and bilateral severe hearing loss. MRI is particularly adept at detecting hypoplasia, agenesis, and associated anomalies of the brainstem auditory circuits (102). The clinical significance of these findings lies in their direct implications for future auditory implant strategies. In individuals with significant structural anomalies of the cochlear nerve, the effectiveness of conventional CI may be limited. Some pediatric patients may require further evaluation for alternative interventions, such as auditory brainstem implantation (ABI). Conversely, for children primarily experiencing conductive or mixed hearing loss alongside specific anatomical anomalies, tailored reconstructive approaches—such as bone-conduction implants—might prove more appropriate (103).

The role of imaging assessments in managing perinatal and NICU-related hearing concerns extends beyond merely enhancing anatomical understanding; it also facilitates the transition from phenotypic characterization of hearing loss to etiological identification. Imaging studies should be a core component of the assessment framework for high-risk children, particularly those with severe or unilateral hearing loss, suspected genetic disorders, or those being considered for cochlear implants. Integrating this information with audiology, genetic testing, and clinical exposure history is crucial. Clear localization of the etiology enables more targeted auditory rehabilitation decisions and long-term prognosis assessments.

Perinatal and NICU-related hearing concerns arise not from a singular event, but from a confluence of various biological and iatrogenic exposures, compounded by developmental immaturity. Prematurity, hypoxia, illness, hyperbilirubinemia, ototoxic medicines, and the degree of circulatory support collectively influence the range of auditory susceptibility in high-risk newborns. For this population, complete screening before discharge is simply the initial step in risk identification. The essential factor is the implementation of a continuous, stratified, and multimodal follow-up system based on exposure intensity and etiological indicators, aimed at early identification and intervention of auditory function abnormalities prior to critical periods for language and neurological development.

## 7 Clinical implications: from etiological recognition to risk-informed surveillance

### 7.1 Interpreting risk indicators beyond the newborn screen

The evidence reviewed above indicates that pediatric hearing loss should not be conceptualized solely as a condition either detected or excluded at birth. UNHS remains indispensable for the early identification of congenital hearing loss. However, it remains a cross-sectional assessment performed at a single developmental time point. Its performance is limited for conditions that emerge after the neonatal period, progress gradually, fluctuate over time, or primarily affect neural synchrony rather than cochlear outer hair cell function (6, 104). This limitation is particularly relevant for children with cCMV infection, pathogenic or likely pathogenic deafness-related variants, a positive family history, NICU exposure, or syndromic features (105–107).

A central implication is that risk indicators should be interpreted as signals of future auditory vulnerability rather than as established hearing loss (108). Some indicators, such as confirmed congenital infection or biallelic pathogenic variants in well-characterized genes, may provide relatively direct etiological information. Others, including prematurity, low birth weight, hypoxia, hyperbilirubinemia, sepsis, or exposure to ototoxic medication, often function as components of a broader vulnerability profile. Their significance lies not only in their individual associations with hearing loss but also in their potential to interact with developmental immaturity, genetic susceptibility, and postnatal exposures.

This distinction is important because it helps to eliminate two common interpretive errors: false reassurance after a normal newborn screen and overmedicalization of infants who carry risk markers but have no current audiological abnormality (108). A balanced approach requires clinicians and researchers to distinguish confirmed disease, probable etiology, susceptibility, and contextual risk. In this framework, the value of risk indicators is not to replace audiological diagnosis, but to identify children for whom a single normal screening result may be insufficient.

### 7.2 Linking etiology, phenotype, and audiological assessment

Etiological information is most useful when interpreted alongside clinical phenotype and age-appropriate audiological assessment. Genetic forms of hearing loss illustrate this principle clearly. GJB2-related hearing loss may span from congenital severe impairment to mild, delayed-onset, or progressive phenotypes; SLC26A4-related disease is frequently associated with enlarged vestibular aqueduct and fluctuating or progressive hearing loss; OTOF-related disease may present as ANSD with preserved OAE but abnormal ABR; and mt-RNR1 variants primarily indicate susceptibility to aminoglycoside-induced ototoxicity (109, 110). These examples illustrate that genetic findings should not be interpreted as uniform predictors of severity, but as context-dependent markers that require phenotypic correlation.

Infectious etiologies also necessitate longitudinal interpretation. cCMV is particularly significant as many affected infants are asymptomatic at birth and may pass newborn hearing screenings while remaining at risk for delayed-onset, progressive, or fluctuating SNHL (106). Unlike stable congenital hearing loss, cCMV-related auditory impairment may evolve non-linearly, vary between ears, and interact with broader neurodevelopmental outcomes. Thus, the clinical significance of cCMV rests not only in its role as an etiological diagnosis but also in its capacity to identify children whose auditory trajectory may remain uncertain following the neonatal period.

Perinatal and NICU-related exposures further illustrate the necessity of linking risk indicators with lesion-level phenotypes. Severe hyperbilirubinemia, hypoxia, infection, mechanical ventilation, and ototoxic drug exposure can impact the cochlea, auditory nerve, brainstem auditory pathways, or central auditory networks (107, 111). Some children may exhibit threshold elevation, while others may demonstrate neural conduction abnormalities or disproportionate difficulties in speech perception. Consequently, relying on a single test modality may prove inadequate for certain high-risk groups. OAE, ABR, immittance measures, ASSR, behavioral audiometry, and speech perception testing each provide distinct information, with their relevance varying by age, suspected lesion level, and developmental stage (112–114).

### 7.3 Implications for risk-based follow-up

The findings summarized in this review advocate for a risk-based approach to post-screening surveillance, with careful definition of its purpose (104, 106–108). This approach should not be interpreted as a universal mandate for intensive follow-up for all infants or as a rigid management algorithm. Instead, it should be recognized as a principle aimed at aligning the intensity of surveillance with the strength, specificity, and persistence of risk indicators. Children with confirmed etiological diagnoses, such as cCMV, well-characterized pathogenic variants, or syndromic features, may necessitate closer developmental observation compared to those with isolated, weak, or transient risk markers (106).

Risk-based follow-up must also account for developmental timing. Delayed-onset and progressive hearing loss can manifest clinically during infancy, toddlerhood, preschool years, or even later in childhood (108). These critical periods coincide with rapid language development, social communication, and early learning. Consequently, surveillance strategies should not only consider auditory thresholds but also incorporate parental concerns, speech-language milestones, listening behaviors, and functional communication. In cases of suspected ANSD, unilateral hearing loss, or central auditory involvement, speech perception and real-world listening challenges may provide more insightful information than pure-tone thresholds alone (112–114).

Risk-based surveillance at the level of public health should be feasible, proportionate, and fair (115, 116). Systems that identify more at-risk infants without confirming a diagnosis will increase the demand for healthcare services due to poor communication with parents and a failure to intervene. Failure to consider the risk index may lead to the progression of undetected, slow-onset hearing loss and thus miss the window for intervention. Based on the above review, not all children need to be screened frequently; rather, according to their reasons for needing screening and any other risk factors, some children may require more often.

## 8 Evidence gaps and future directions

### 8.1 Clarifying causality and risk magnitude

Although many etiologies and risk indicators for pediatric hearing loss have been identified, the strength of evidence varies substantially across domains. Genetic causes, cCMV, severe hyperbilirubinemia, ototoxic exposure, and structural inner ear abnormalities have relatively clear mechanistic or clinical links to childhood hearing loss (111). In contrast, evidence for several prenatal environmental, metabolic, immune, paternal, and epigenetic exposures remains less definitive (87–89). These factors are biologically plausible and may contribute to auditory vulnerability, but their independent effects are difficult to isolate because they often co-occur with socioeconomic disadvantage, maternal illness, prematurity, infection, or healthcare access barriers. Specifically, elevated serum uric acid and aberrant uric acid metabolism may contribute to such metabolic pathways of hearing impairment, potentially through the induction of oxidative stress and microvascular dysfunction in the cochlea. Furthermore, in certain pediatric hereditary or complex disorders, aberrant uric acid metabolism frequently co-occurs with auditory dysfunction.

Future studies should move beyond simple exposure–outcome associations and clarify causal pathways, dose–response relationships, timing of vulnerability, and interactions among multiple risks. Prospective birth cohorts with repeated audiological assessment, standardized exposure measurement, genomic or molecular data, and developmental follow-up would be particularly valuable. Such designs could help distinguish factors that directly cause hearing loss from those that function primarily as markers of broader developmental risk. This distinction is essential for developing clinically meaningful risk stratification systems.

### 8.2 Improving genotype–phenotype and etiology–phenotype correlation

A second major gap concerns the relationship between molecular or etiological findings and clinical phenotype. Genetic testing has improved diagnostic yield, but interpretation remains challenging when variants have incomplete penetrance, variable expressivity, uncertain pathogenicity, or age-dependent effects (117, 118). The same gene may be associated with different degrees of severity, timing of onset, progression, and treatment response. Similarly, cCMV-related hearing loss may be unilateral or bilateral, stable or fluctuating, congenital or delayed-onset, whereas NICU-related hearing loss may involve cochlear, neural, or central mechanisms (111).

Future research should prioritize longitudinal genotype–phenotype and etiology–phenotype mapping. Studies should report not only whether hearing loss is present, but also its age of onset, laterality, progression, audiometric configuration, speech perception profile, electrophysiological features, imaging findings, and developmental outcomes. This level of phenotypic detail is necessary to identify which children are most likely to benefit from extended surveillance and which markers have limited predictive value. Avoiding both under-recognition of delayed-onset disease and unnecessary medicalization of low-risk children is essential.

### 8.3 Evaluating screening expansion, implementation feasibility, and equity

Advances in genetic testing and pathogen detection have enhanced interest in combining UNHS with molecular or infectious screening (119–122). Such approaches may improve identification of infants at risk for delayed-onset or progressive hearing loss, particularly in the context of cCMV or pharmacogenetic susceptibility. However, expanded screening raises important questions about cost-effectiveness, informed consent, variant interpretation, parental anxiety, follow-up capacity, and equitable access (123–126). Higher diagnostic yield does not directly translate into better child outcomes if health systems cannot provide timely confirmation, counseling, monitoring, and intervention.

Considering this, future evaluations should assess screening strategies using established implementation and outcome indicators rather than a separate public health assessment matrix. Relevant indicators may include screening coverage, referral completion, diagnostic confirmation, loss to follow-up or loss to documentation, age at diagnosis, age at intervention, access to audiological and rehabilitation services, family understanding of results, cost-effectiveness, and disparities by socioeconomic status, geography, ethnicity, language, and healthcare access (125, 126). This is particularly important in resource-restrained settings, where the most effective strategy may not be the most technologically advanced, but the one that consistently links identification with follow-up and intervention. Equity should be treated as a core dimension of screening effectiveness rather than a secondary implementation concern.

### 8.4 Toward developmentally informed surveillance

The broader challenge for the field is to develop surveillance models that acknowledge the developmental nature of childhood hearing loss. Many clinically significant forms of hearing impairment do not remain static at birth; they may emerge, progress, fluctuate, or only become functionally apparent when listening demands increase (112–114). Future research should include repeated hearing assessments, speech-language outcomes, caregiver-reported listening behaviors, and neurodevelopmental measures to better reflect the real-world impact of mild, unilateral, neural, or progressive hearing loss.

A developmentally informed research agenda must also integrate data across various disciplines. Fields such as genetics, infectious disease, neonatology, audiology, otolaryngology, speech-language pathology, epidemiology, and public health each offer distinct perspectives (115). The next generation of evidence will require not only improved testing but also better integration of etiological information, clinical phenotype, developmental trajectory, and health system context. Such integration is essential for transitioning from early detection to early recognition of children whose auditory development may remain at risk over time.

## 9 Conclusion

The UNHS era has substantially improved the early detection of congenital hearing loss, revealing the complexity of pediatric hearing loss that cannot be fully addressed by a single neonatal screening event. Mild, unilateral, delayed-onset, progressive, and neural forms of hearing loss may escape early detection, and some children who pass newborn screening remain at risk due to genetic susceptibility, congenital infection, intrauterine exposures, NICU-related complications, or syndromic conditions. This review highlights the need to distinguish among etiological causes, risk indicators, and clinical phenotypes. Genetic variants, cCMV, structural inner ear abnormalities, ototoxic susceptibility, and NICU-related exposures differ in mechanism, predictive value, and clinical implications. Likewise, audiological phenotypes vary according to onset timing, lesion level, progression, laterality, and functional impact. Recognizing these distinctions is critical for interpreting risk following UNHS and for identifying children whose hearing and communication development may warrant closer observation. The central implication is that childhood hearing loss should be viewed as a developmental condition characterized by heterogeneous causes and trajectories, rather than as a fixed diagnosis established solely at birth. Together, etiological knowledge, risk indicators, and age-appropriate audiological assessments provide a stronger foundation for recognizing delayed-onset and progressive hearing loss. Future research should refine risk estimates, clarify causal pathways, enhance genotype–phenotype correlations, and evaluate equitable approaches to post-screening surveillance. By aligning etiological understanding with developmental follow-up, pediatric hearing health systems may decrease missed or delayed diagnoses and improve long-term language, educational, and psychosocial outcomes.
