# Supplementary material for: Pediatric hearing loss in the universal newborn hearing screening era: etiologies, risk indicators, and clinical phenotypes
Source: Front Public Health. 2026 Sep 24;14:1836340. doi: 10.3389/fpubh.2026.1836340 (PMC13647992; doi:10.3389/fpubh.2026.1836340)
Supplement: Supplementary file 1 [file Image_1.pdf]

Supplementary Material

1 Supplementary Figures

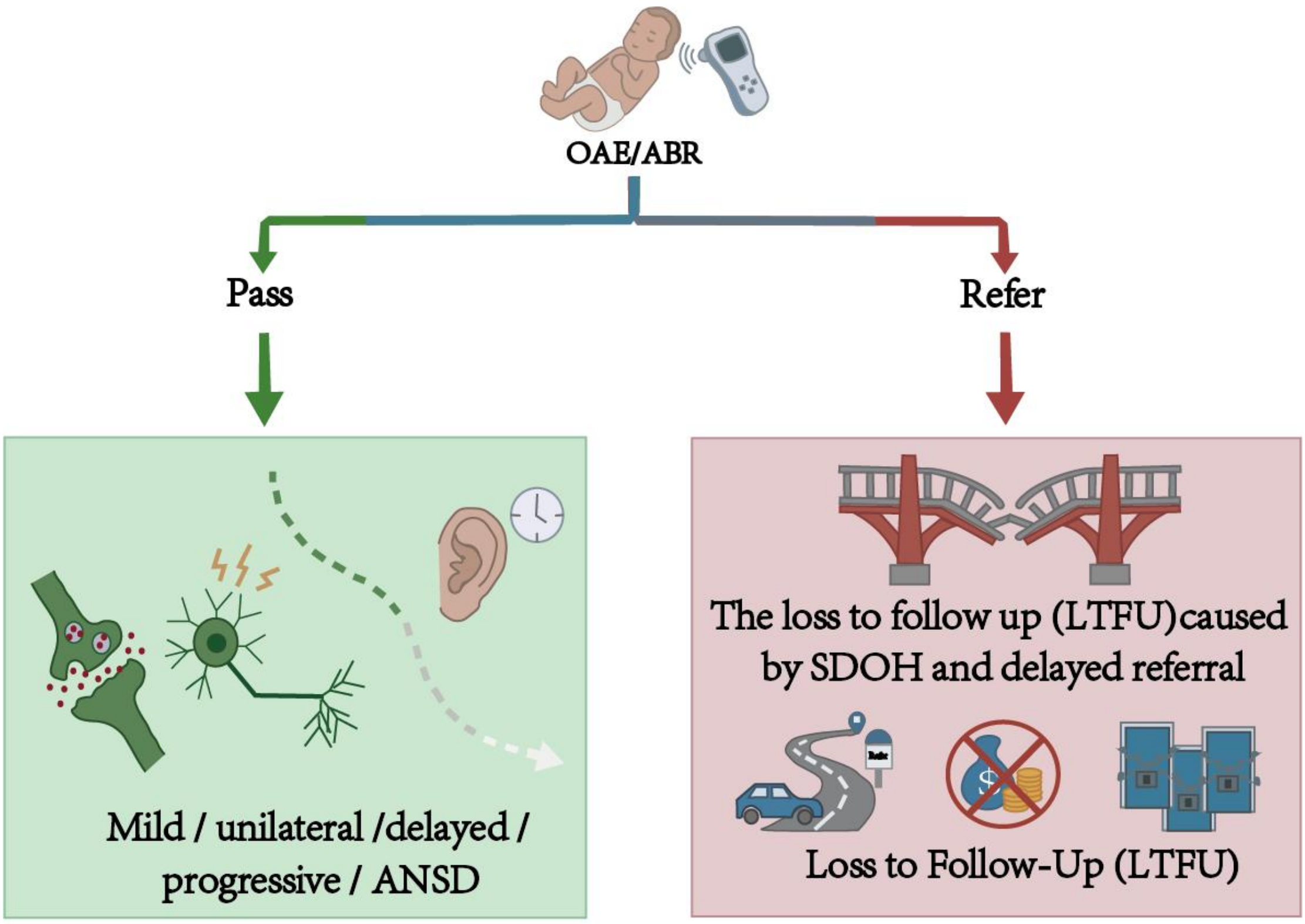

Supplementary Figure 1. Limitations of universal newborn hearing screening in detecting heterogeneous pediatric hearing loss. Universal newborn hearing screening (UNHS) employing otoacoustic emissions and/or automated auditory brainstem response has significantly enhanced early identification of congenital hearing loss. However, a single neonatal screening episode may fail to detect all clinically relevant forms of pediatric hearing loss. Mild, unilateral, delayed-onset, progressive, and certain neural forms of hearing impairment, including auditory neuropathy spectrum disorder, may be overlooked or become evident only later in infancy or childhood. Additionally, issues such as incomplete referrals, delayed diagnostic confirmation, and loss to follow-up can diminish the effectiveness of post-screening care pathways.

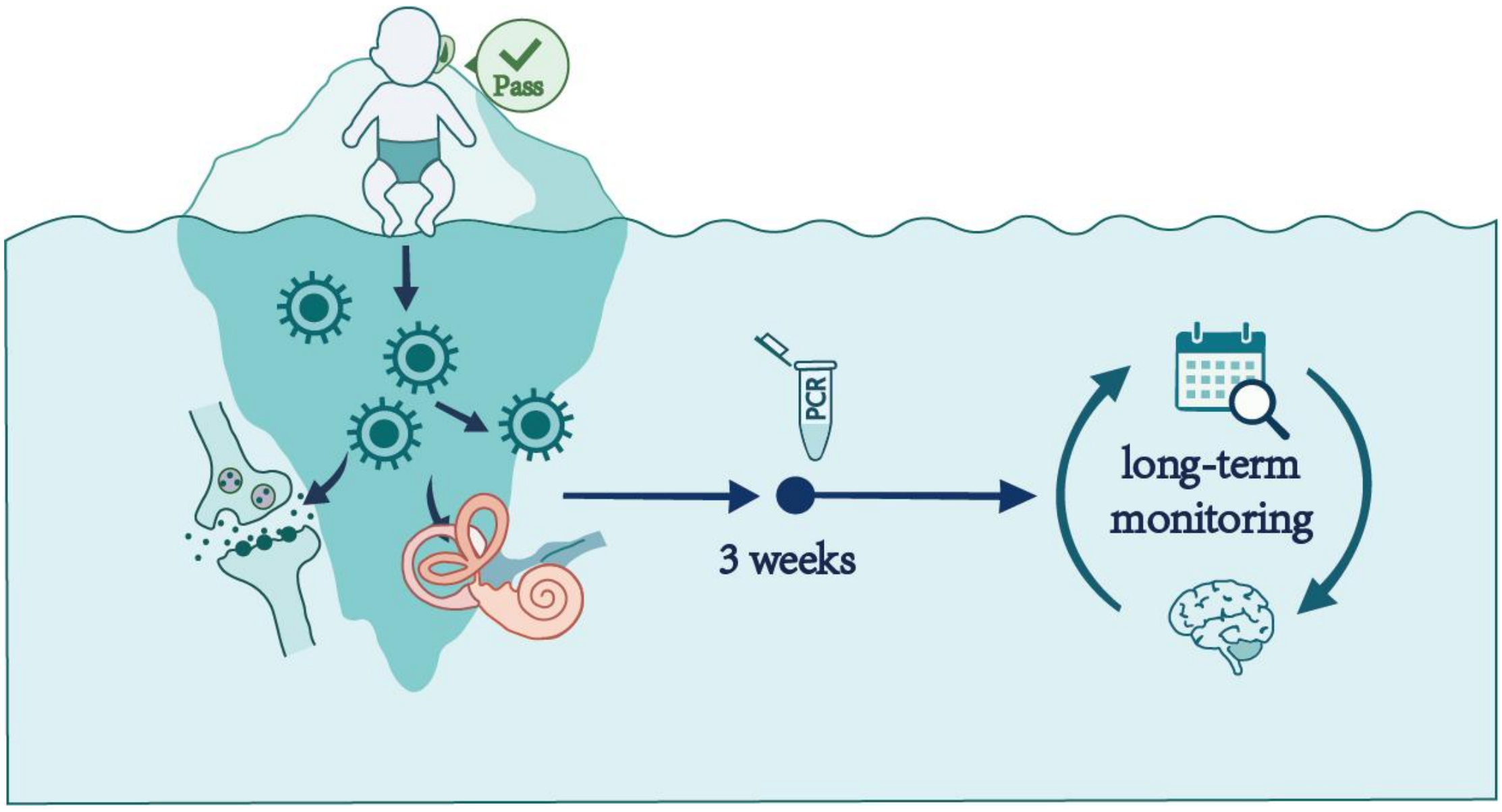

Supplementary Figure 2. Congenital cytomegalovirus infection and delayed-onset pediatric hearing loss. Congenital cytomegalovirus (cCMV) infection represents a significant non-genetic cause of pediatric sensorineural hearing loss. Many affected infants are asymptomatic at birth and may pass newborn hearing screening while still being at risk for delayed-onset, progressive, fluctuating, unilateral, or bilateral hearing loss. Virological testing, utilizing saliva or urine polymerase chain reaction within the first three weeks of life, is crucial for differentiating congenital from postnatal infection. Thus, cCMV highlights the importance of etiological information in assessing auditory risk following a normal newborn hearing screen.

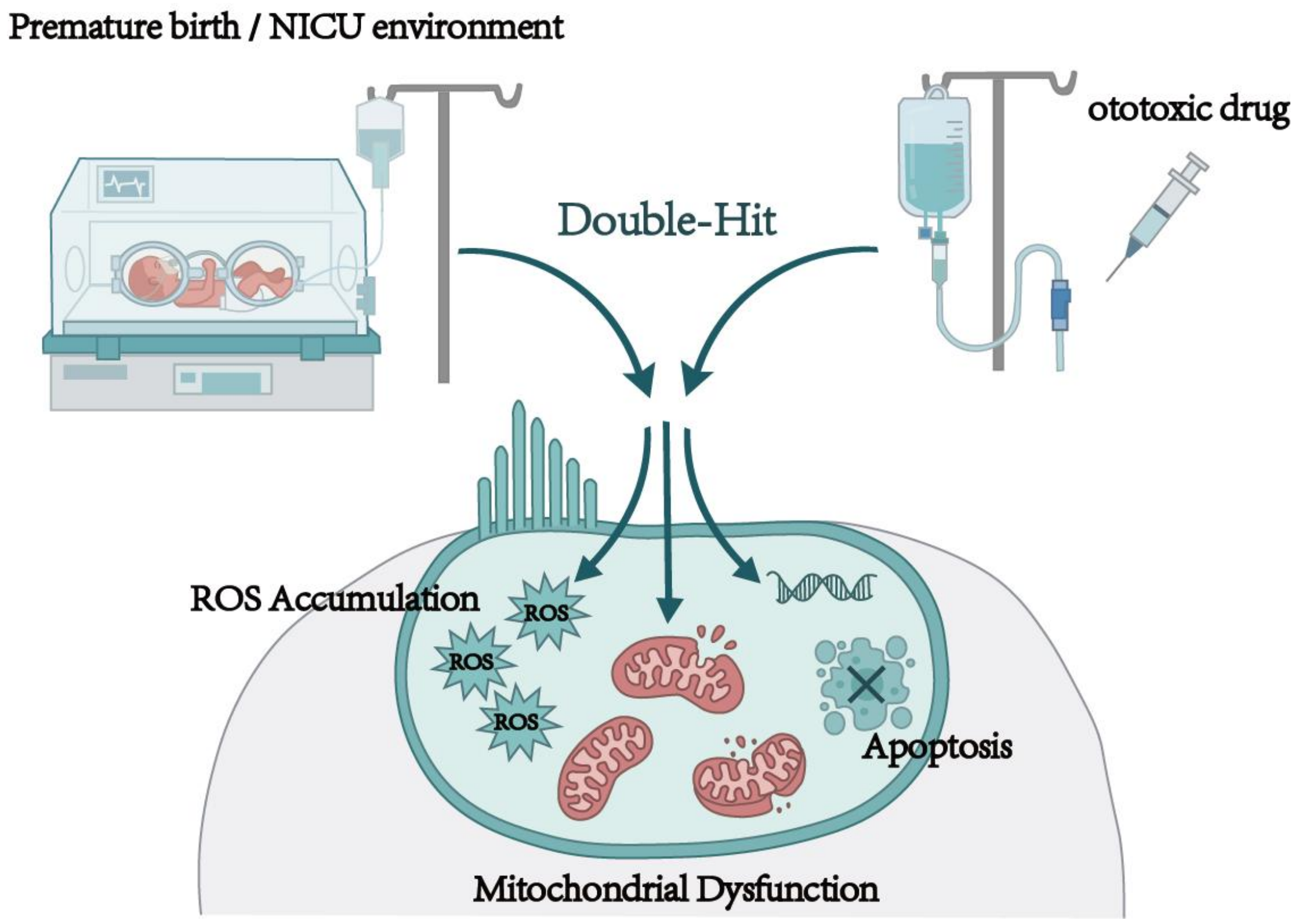

Supplementary Figure 3. Drug-induced ototoxicity and auditory vulnerability in the neonatal intensive care unit environment. Drug-induced ototoxicity in neonatal intensive care unit settings may result from interactions among developmental immaturity, critical illness, pharmacological exposure, and genetic susceptibility. Factors such as prematurity, hypoxia, infection, unstable perfusion, and metabolic disturbances can heighten the vulnerability of the cochlea, auditory nerve, and brainstem auditory pathways. Exposure to aminoglycosides, loop diuretics, and other potentially ototoxic medications may further exacerbate auditory injury through mechanisms including oxidative stress, mitochondrial dysfunction, hair cell damage, and microcirculatory disruption. Additionally, variants in mt-RNR1 may increase susceptibility to aminoglycoside-associated hearing loss.

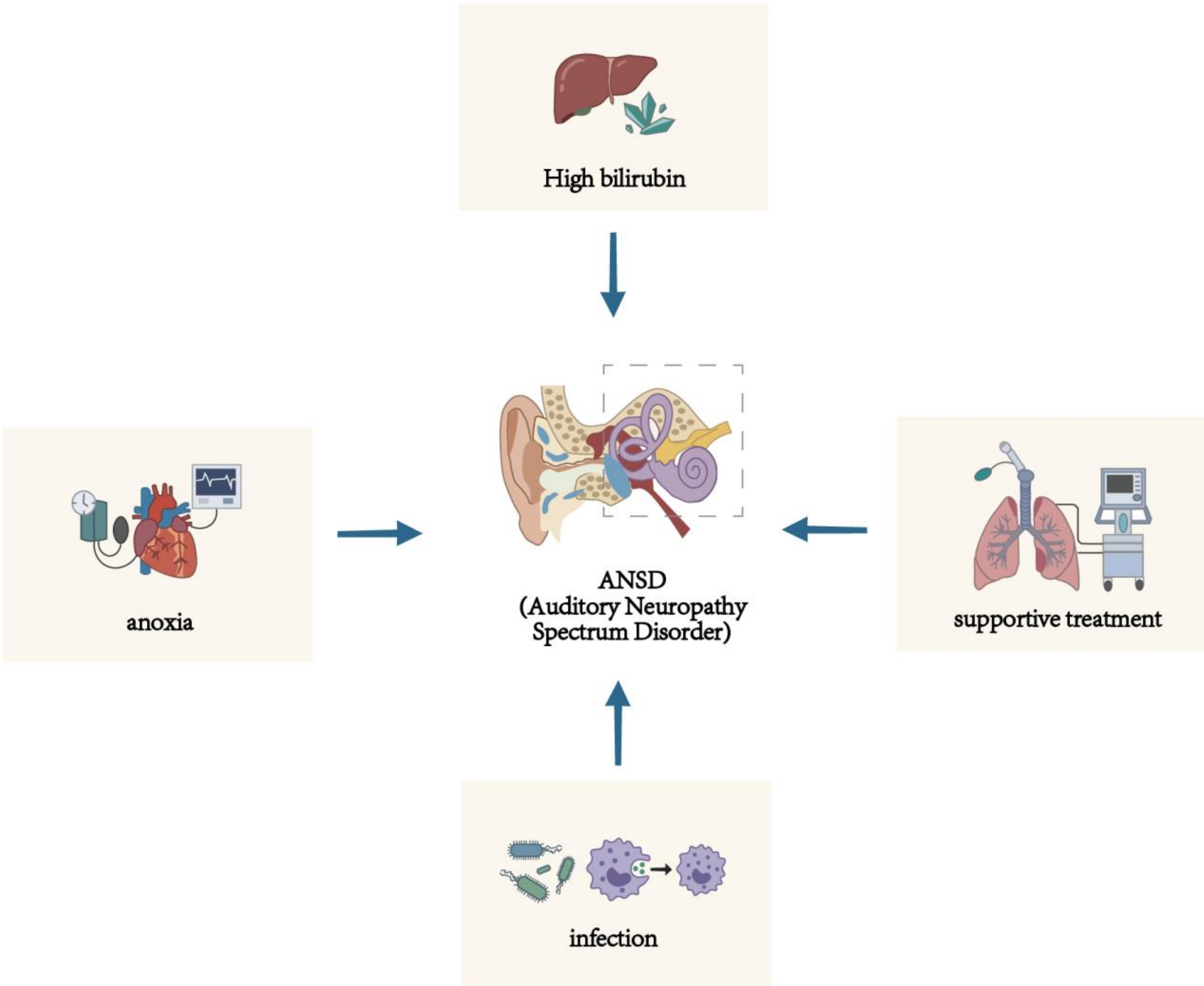

Supplementary Figure 4. Multifactorial neonatal intensive care unit exposures and auditory pathway vulnerability. Hearing loss among infants exposed to the neonatal intensive care unit often results from a combination of biological and iatrogenic risk factors rather than from a single isolated cause. Severe hyperbilirubinemia, hypoxia, sepsis, hemodynamic instability, mechanical ventilation, and exposure to supportive or ototoxic medications can impact various levels of the auditory pathway. Hyperbilirubinemia is closely linked to auditory nerve and brainstem involvement, while hypoxia and hypoperfusion may compromise cochlear microcirculation and metabolic homeostasis. Additionally, systemic inflammation and sepsis can further affect auditory neural pathways and broader neurodevelopmental outcomes.

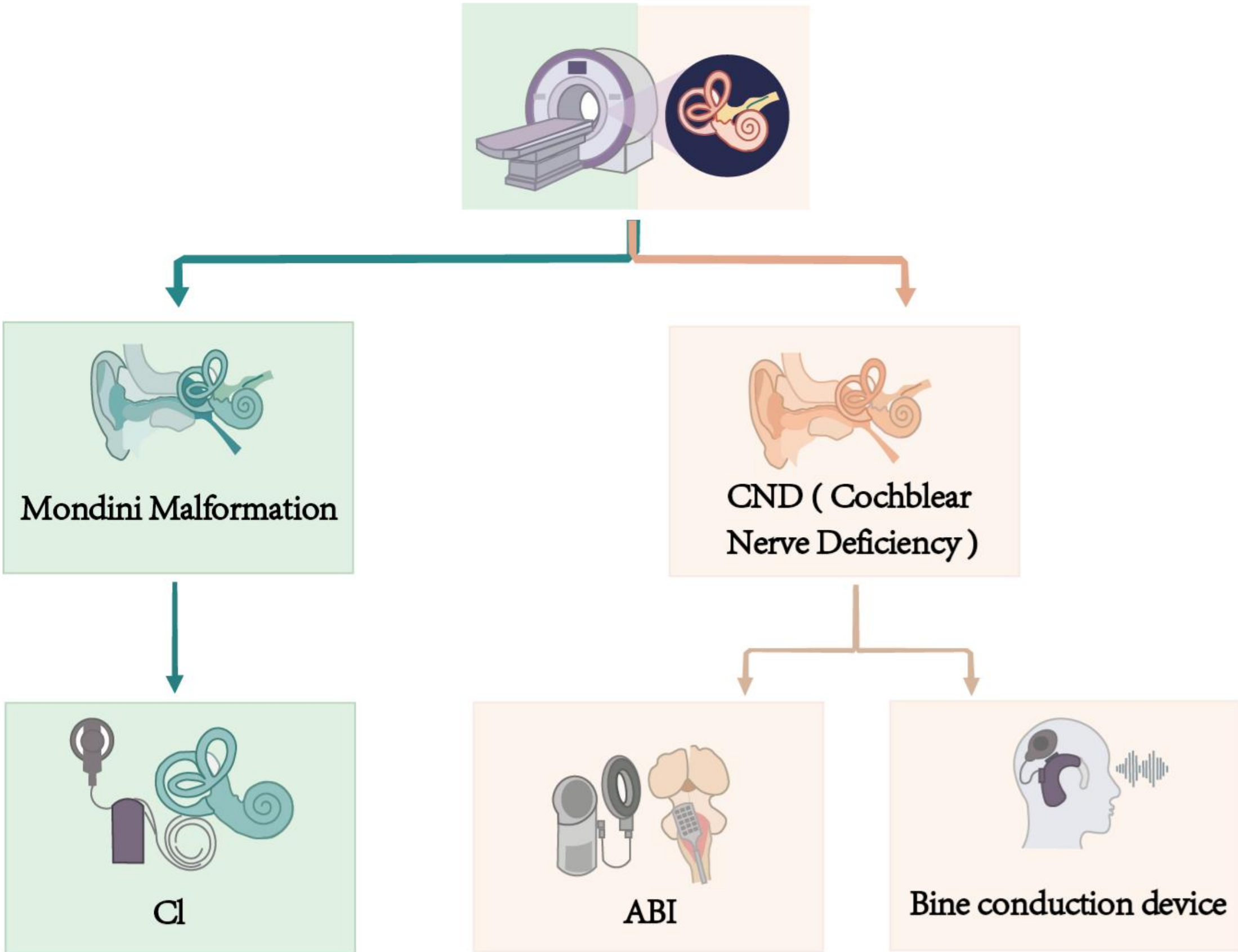

Supplementary Figure 5. Imaging assessment and anatomical etiologies of pediatric hearing loss. High-resolution computed tomography (CT) and magnetic resonance imaging (MRI) can offer complementary anatomical information for selected children with pediatric hearing loss. Imaging can aid in identifying inner ear malformations, enlarged vestibular aqueducts, cochlear nerve deficiencies, and other structural abnormalities that contribute to congenital, unilateral, severe, or syndromic hearing loss. Anatomical findings can enhance etiological interpretation and provide context for audiological phenotypes, particularly when electrophysiological or behavioral test results do not align with the clinical presentation.
